# High-Resolution Cartography of the Transcriptome and Methylome Landscapes of Diffuse Gliomas

**DOI:** 10.3390/cancers13133198

**Published:** 2021-06-26

**Authors:** Edith Willscher, Lydia Hopp, Markus Kreuz, Maria Schmidt, Siras Hakobyan, Arsen Arakelyan, Bettina Hentschel, David T. W. Jones, Stefan M. Pfister, Markus Loeffler, Henry Loeffler-Wirth, Hans Binder

**Affiliations:** 1 IZBI, Interdisciplinary Centre for Bioinformatics, Universität Leipzig, Härtelstr. 16-18, 04107 Leipzig, Germany; edith.willscher@uk-halle.de (E.W.); lydia.hopp@gmx.net (L.H.); schmidt@izbi.uni-leipzig.de (M.S.); 2 IMISE, Institute for Medical Informatics, Statistics and Epidemiology, Universität of Leipzig, Härtelstr. 16-18, 04107 Leipzig, Germany; markus.kreuz@izi-extern.fraunhofer.de (M.K.); bettina.hentschel@iimise.uni-leipzig.de (B.H.); markus.loeffler@imise.uni-leipzig.de (M.L.); 3 Research Group of Bioinformatics, Institute of Molecular Biology of the National Academy of Sciences of the Republic of Armenia, 7 Hasratyan Str., Yerevan 0014, Armenia; sirashakobyan@gmail.com (S.H.); aarakelyan@sci.am (A.A.); 4 Armenian Bioinformatics Institute (ABI), 7 Hasratyan Str., Yerevan 0014, Armenia; david.jones@dkfz.de (D.T.W.J.); s.pfister@dkfz.de (S.M.P.); 5 Hopp Children’s Cancer Center Heidelberg (KiTZ), Im Neuenheimer Feld 430, 69120 Heidelberg, Germany

**Keywords:** grade II–IV gliomas, gene expression, DNA methylation, tumor heterogeneity, molecular subtypes, tumor evolution, self-organizing maps machine learning, integrative bioinformatics

## Abstract

**Simple Summary:**

A high degree of molecular heterogeneity is a fundamental characteristic of diffuse gliomas, a brain tumor entity, which splits into several subtypes of different but overall adverse prognosis. Heterogeneity is governed by a handful of key mutations—first of all, of the isocitrate dehydrogenase gene. It drastically affects DNA methylation on a genome-wide scale. DNA methylation acts as an important regulator of gene transcription with consequences for glioma physiology. We here present a combined gene expression and DNA methylation study with the focus on lower-grade (II–III), adult-type gliomas. It aimed at deciphering glioma heterogeneity into molecular subtypes at a finer granularity level and at characterizing the underlying modes of gene regulation. Our analysis made use of high-resolution molecular portrayal, a machine learning approach to visualize complex genomic data. The results support the importance of epigenetics for glioma diversity and, in consequence, for prognosis and epigenetics-directed treatment.

**Abstract:**

Molecular mechanisms of lower-grade (II–III) diffuse gliomas (LGG) are still poorly understood, mainly because of their heterogeneity. They split into astrocytoma- (IDH-A) and oligodendroglioma-like (IDH-O) tumors both carrying mutations(s) at the isocitrate dehydrogenase (IDH) gene and into IDH wild type (IDH-wt) gliomas of glioblastoma resemblance. We generated detailed maps of the transcriptomes and DNA methylomes, revealing that cell functions divided into three major archetypic hallmarks: (i) increased proliferation in IDH-wt and, to a lesser degree, IDH-O; (ii) increased inflammation in IDH-A and IDH-wt; and (iii) the loss of synaptic transmission in all subtypes. Immunogenic properties of IDH-A are diverse, partly resembling signatures observed in grade IV mesenchymal glioblastomas or in grade I pilocytic astrocytomas. We analyzed details of coregulation between gene expression and DNA methylation and of the immunogenic micro-environment presumably driving tumor development and treatment resistance. Our transcriptome and methylome maps support personalized, case-by-case views to decipher the heterogeneity of glioma states in terms of data portraits. Thereby, molecular cartography provides a graphical coordinate system that links gene-level information with glioma subtypes, their phenotypes, and clinical context.

## 1. Introduction

Diffuse gliomas are a heterogeneous entity of brain cancers with an adverse prognosis. Molecular subtyping based on high-throughput omics technologies has emerged as an important concept to better understand the biology of this disease. Genetic lesions, namely, the IDH1/2 mutation (IDH-mut, for glossary, see Abbreviations) in combination with co-deletions of chromosomes 1 and 19 (Chr.1p19q codel) presently provide the major outcome- and treatment-relevant molecular glioma markers [1,2]. IDH-mut gliomas show drastically changed DNA methylation patterns compared with IDH-wt gliomas overall subsumed as GCIMP (glioma CPG island methylator phenotype). A number of studies have analyzed alterations of DNA methylation in gliomas ‘in space and/or time’ [3,4,5,6,7,8,9,10,11] and developed classification tools for brain tumor entities [12]. DNA methylation acts as an important regulator of gene transcription with consequences for gliomas physiology [13,14] in the context of immune editing and escape related to the tumor microenvironment, of mitotic activity, glioma development, and of treatment resistance on cell and gene levels [10,15,16,17]. Aberrant epigenomes define adult brain cancers, as demonstrated by widespread changes to DNA methylation patterns, redistribution of histone methylation marks, and disruption of chromatin structure, demonstrating the impact of genetic, metabolic, and microenvironmental factors on mechanisms of epigenetic deregulation [18]. In general, DNA methylation better memorizes cell of origin properties [19] compared with the transcriptome, which more reflects the actual activity state of the (cancer) cells. Hence, a combined view on both gene expression and DNA methylation could further improve our understanding of molecular mechanisms of glioma etiology.

Our previous expression and DNA methylation profiling study of grade II and III lower grade gliomas (LGG) revealed rich heterogeneity in terms of eight transcriptionally and six methylation defined groups that were only partially linked each to another and to the genomic groups [20,21]. We here extended this work with the main aim of better understanding the functional background of the diversity and the mutual relations between gene expression and promoter methylation at the gene level. Of special interest are hereby grade II–III astrocytomas, which are split into three methylation and expression subtypes differing in methylation levels, partly resembling GCIMP-low gliomas, a special subgroup of relatively worse outcome revealing immunogenic tumor microenvironment with possible impact for treatment resistance and tumor recurrence [20,22]. The molecular background of these astrocytoma subtypes is mostly unclear, partly associating with a low methylation GCIMP state resembling grade IV glioblastomas (GBM) [20]. In the first part of this publication, we therefore performed a joint analysis of grade II-III LGG together with grade IV GBM [23] and of grade I pilocytic astrocytomas (PA) in order to find similarities and differences in their expression patterns. PA-like characteristics were previously identified in part of GBMs [22], showing a specific immunogenic signature. The second part focuses on a combined analysis of expression and DNA methylation data of LGG. It aims at extracting modules of co-expressed and co-methylated genes in order to establish a mutual co-regulation network and to characterize their functional impact for glioma development. We address questions such as ‘what drives IDH-mutant LGG to undergo higher-grade transformation and to gain a more aggressive behavior?’, for example, via a hyper-mutator phenotype, changed tumor microenvironment, and/or the loss of methylation in a low GCIMP state [22,24,25].

Previously, we developed the so-called omics ‘portrayal’ method based on self-organizing map (SOM) machine learning [26,27]. It was applied to expression and methylation data of GBM [28,29,30], other cancer entities such as lymphomas [31,32], melanomas [33], and also other diseases [34,35]. SOM portrayal provides molecular maps in terms of a graphical coordinate system that links gene-level information including key genes and co-regulated ‘signature’ gene sets with glioma subtypes, their functional context, and prognosis. We herein applied this high-resolution molecular cartography to the transcriptomes of grade I–IV gliomas and to LGG expression and methylation data for a holistic molecular characterization of this tumor entity.

## 2. Materials and Methods

### 2.1. Patients, Tumors, and Data

**Figure 1:**
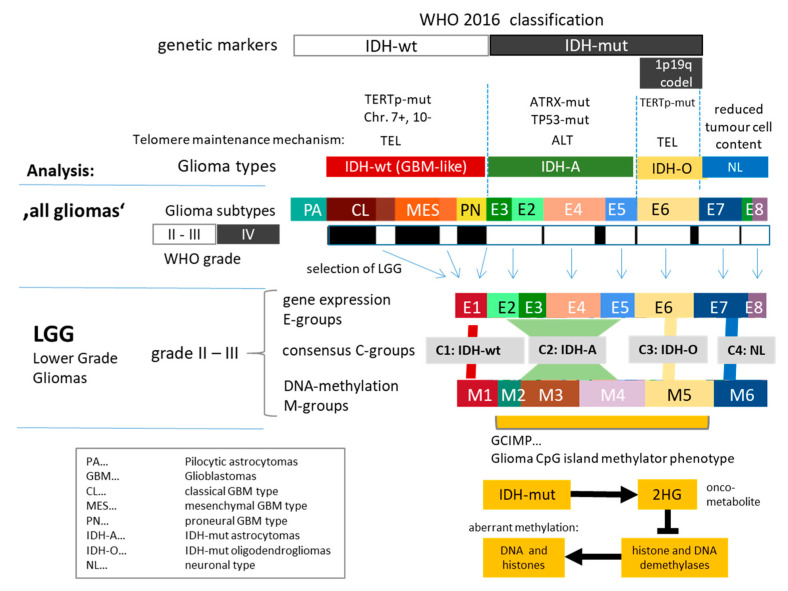
Overview about glioma strata and of their analyses: ‘All glioma’ analysis comprises expression data of pilocytic astrocytomas and of grade II–IV gliomas (LGG and GBM), which were stratified as shown in the figure. Then, combined analysis of expression and DNA methylation data of LGG was performed in order to study the effect of DNA methylation on glioma biology. About 85% of LGG carry mutations in the IDH gene, which causes aberrant methylation of DNA and histone side chains via repression of demethylating enzymes by the onco-metabolite 2 hydroxy-glutamate (2HG). E-groups and M-groups of LGG were stratified strictly on the basis of gene expression and DNA methylation data, respectively (see [20]), which, in consequence, gives rise to only partly matched E- and M-groups as well as genetic WHO-classes based on IDH mutation and chromosome 1p and 19q co-deletion status. Abbreviations and color coding of the groups are used throughout the paper.

We here studied WHO grade II and III adult-type gliomas (lower-grade gliomas, LGG) collected from 137 patients who were previously analyzed by microarray-based gene expression profiling and candidate gene analyses [21]. A total of 122 of the tumors were also characterized using array-based DNA methylation profiling (Illumina 450 K arrays) [20]. LGG were stratified into eight expression types (E1–E8) and, independently, into six methylation types (M1–M6) [20] (Figure 1). Expression data of grade IV glioblastomas (GBM) together with their classification into classical (CL), mesenchymal (MES), and proneural (PN) tumors according to Verhaak’s stratification [36] were taken from [23] (94 tumors) and resorted into IDH-wt and IDH-mut groups (see below). The classifications were previously verified by TCGA (The Cancer Genome Atlas) data comprising GBM and LGG [20,21,23]. Gliomas were complemented by gene expression data of 16 pilocytic astrocytomas kindly provided by the German Glioma Network consortium (GGN). For association of telomere length with expression data and mutation counts, we made use of the TCGA cohort stratified according to our subtypes as described previously [20]. Corresponding telomere length data were taken from [22], and mutations were downloaded from the TCGA data portal (https://registry.opendata.aws/tcga/; accessed date: 10 October 2020).

### 2.2. Expression and DNA Methylation Analyses

Expression and methylation data were processed as described in [20]. In short, gene expression data were calibrated, transformed into log_10_-scale, quantile-normalized, and corrected for background noise. CpG DNA methylation beta values (defined as fractional methylation) were mapped to the promoter region of each gene ranging from 2 kb upstream to 200 bp downstream of the transcription start site of each gene using RefSeq mRNA annotation and then averaged over all included CpG to obtain one mean methylation beta value for each gene promoter. Genes located on chromosomes X and Y were excluded from analyses to avoid a gender bias. Hence, we applied a gene-centric analysis of CpG methylation values for direct comparison with gene expression values and for consistency with our previous methylation analyses in grade IV glioma [20,28,29]. Comparison with specific marker CpGs and CpG sets taken from the literature in our dataset, e.g., to differentiate between methylation states in the subtypes, was performed previously [20].

### 2.3. Data Portrayal Using Self-Organizing Maps

After pre-processing, expression and DNA methylation data were clustered using self-organizing map (SOM) machine learning. The SOM portrayal method transforms the gene-centric data into metagene profiles of reduced dimensionality and visualizes them as two-dimensional quadratic portrait-images [27]. For expression and methylation data, two separate SOM were trained independently with 50 × 50 and 30 × 30 metagene resolution, respectively. Subtype-specific mean portraits were generated by averaging the metagene landscapes of all cases belonging to one class. Difference portraits between subtypes were calculated as the differences between the metagene values in each pixel of the maps. Details of SOM training and parametrization were described previously [26,27]. Bioinformatics downstream analyses including class discovery and sample diversity analysis, feature selection in terms of so-called spots, and knowledge mining using gene sets was performed as described in [27,37]. Association with phenotypes using correlative ‘phenotype portraits’ was described in [31]. All downstream methods are implemented in the R-package ‘oposSOM’ [38] applied for analysis. 

### 2.4. oposSOM Browser

Results of transcriptome and methylome analyses presented in this publication can be interactively discovered regarding further details using the oposSOM browser [39] available in the internet via the IZBI portal (https://www.izbi.uni-leipzig.de/opossom-browser/). For details, see data availability statement below.

## 3. Results

### 3.1. Cartography of the Transcriptomic States of PA, LGG, and GBM 

Diffuse ‘lower-grade’ (WHO grade II–III) adult-type gliomas (LGG) share expression signatures derived independently from grade IV glioblastomas (GBMs) [20]. To establish mutual similarities between LGG and GBM and also of grade I pilocytic astrocytomas (PA), we trained a self-organizing map (SOM) of the transcriptomes of, in total, 248 glioma specimens of these three entities. The 16 PAs were considered as a separate group, while the 137 LGGs and 94 GBMs were classified in accordance with [20,21] and [23] (Figure 1). In particular, gliomas lacking the IDH1/2 mutation (IDH-wt) of all grades II–IV were typed according to Verhaaks’ classification into classical (CL), mesenchymal (MES), proneural (PN), and neuronal (NL) gliomas [23,36]. IDH1/2-mutated gliomas (IDH-mut) were divided into classes E2–E6 in accordance with [20] and the recommendation to consider them separately from IDH-wt (GBM) as IDH-A (IDH-mutated astrocytomas) grade IV [40]. The groups E2–E5 assign astrocytoma-like (IDH-A) gliomas mostly lacking the chromosome 1p19q co-deletion, while E6 strongly accumulated chromosome 1p19q co-deleted oligodendrioglioma-like (IDH-O) tumors. The two NL subtypes E7 and E8 collect samples with reduced tumor cell content independent of the IDH-mut status, showing partly healthy brain transcriptome characteristics [20]. E8 appears as a mixture of E7 and E3. E4 was shown to express an astrocytic signature, while E2 and E3 were assigned to more metabolically active and inflammatory subtypes, respectively [20]. 

**Figure 2:**
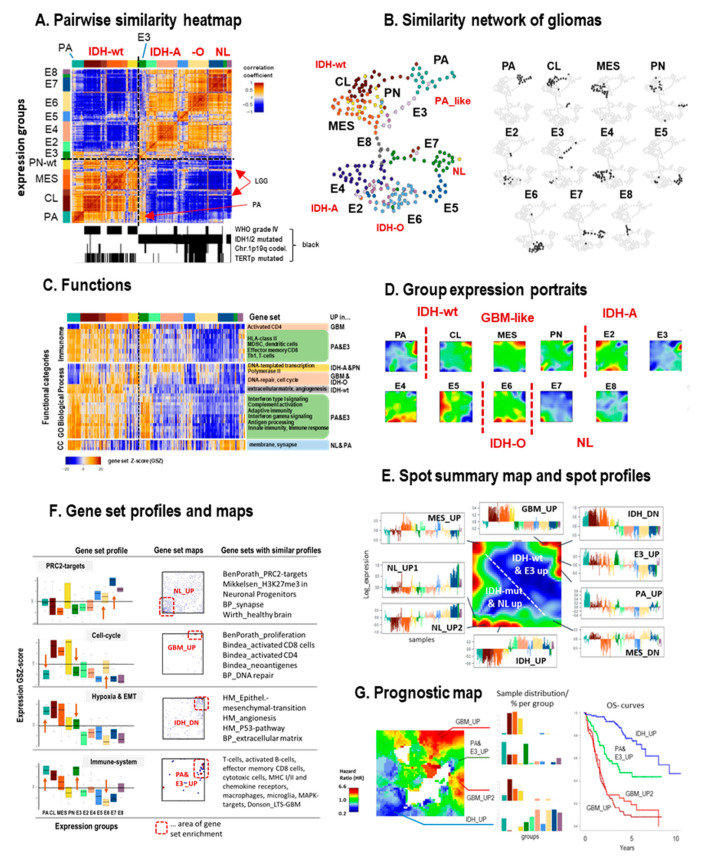
Cartography of ‘all-glioma’ transcriptomes considering pilocytic astrocytomas (PA) and IDH-wt, IDH-A, IDH-O, and neuronal (NL) gliomas of WHO-grade II- IV (see text): (**A**) The pairwise similarity heatmap reveals two major clusters formed by IDH-wt and IDH-mut (and NL) gliomas, respectively. The IDH-mut subgroup E3 reveals similarities with PA and, to a lesser degree, also with CL and MES IDH-wt (GBM-like) gliomas. (**B**) The similarity net visualizes mutual similarities between glioma specimen (dots). It separates virtually all subgroups (PA, CL, MES, and PN for IDH-wt and E2–E8 for IDH-mut). In the right part, samples of each group are separately colored in black. (**C**) Heatmap of expression profiles of selected gene sets indicate up- and downregulation in a subtype-specific fashion (see Supplementary File 1: Figures S1–S4 for details). (**D**) The SOM maps of the different expression groups ‘portrait’ their expression patterns in terms of up- (in red) and downregulated (in blue) gene clusters. Note that each group is characterized by a unique ‘fingerprint’ expression portrait. (**E**) The overexpression summary map provides an overview about the spots upregulated in any of the groups, as illustrated by selected expression profiles. They indicate upregulation of the respective genes in a subtype-specific fashion. For example, ‘E3_UP’ assigns a spot that specifically upregulates in group E3 and partly in PA (see also the group portraits of E3 and PA in part D). (**F**) Box plots of the group-related expression of selected gene sets reveal characteristic effects. While PRC2 targets (and healthy brain functions) loose expression in gliomas compared with the NL subtype, cell cycle activity, immune response, and hypoxia (and EMT, epithelial mesenchymal transition) functionalities gain in expression, especially in IDH-wt gliomas. Interestingly, PA and E3 concertedly change in virtually all situations (see arrows). The gene set maps indicate accumulation of the genes of the respective set (shown by dots) in distinct areas of the map. Gene sets were taken from the literature [41,42,43,57]. (**G**) The prognostic map colors areas in which upregulation of the respective genes associates with high (red) or low (blue) hazard ratio (HR). The barplots show the composition of groups expressing the respective genes together with the respective overall survival curves (see Figure S7 for details).

The pairwise similarity heatmap reveals two major clusters referring to gliomas with and without mutations of the IDH1/2 gene (Figure 2A). This bipartite pattern simply reflects the strong effect of aberrant DNA methylation on the transcriptomes of IDH-mut tumors (see below and [22]). Specifics of the different groups become evident as brown quadratic clusters along the diagonal of the heatmap and as well separated sample ‘clusters’ in the similarity network (Figure 2B). Interestingly, IDH-mut gliomas of E3 reflect similarities with IDH-wt tumors, especially with PA and, to a lesser degree, with the MES and CL groups. Gene set analysis shows that a series of immunity-related functions activate in PA and E3 (and, to a lesser degree in CL and MES) but are downregulated in IDH-O/E6, while proliferation-related functions are upregulated in GBM-like (and, to a lesser degree in IDH-O) gliomas (Figure 2C and Supplementary File 1: Figures S1–S4 for details).

SOM portrayal visualizes the specific expression landscapes of the glioma groups in terms of red and blue spot-like clusters of co-regulated genes (Figure 2D and Figure S5), which were summarized in the overexpression summary map together with the associated expression profiles (Figure 2D). Gene sets underpin the functional context of the spots (Figure 2E). The expression of targets of the polycomb repressive complex 2 (*PRC2*, [41]) strongly decays in LGG and especially in IDH-wt GBM compared with the NL gliomas, which reflects the loss of healthy brain function associated with the progressive de-repression of developmental functions and de-differentiation of neuronal cellular programs in gliomas [28]. *PRC2* target genes accumulate in the area of the NL_UP1 spot, thus illustrating the relation between expression portraits and profiles (see the gene set map in Figure 2D). Other spots contain genes specifically upregulated in IDH-mut or in different IDH-wt gliomas (MES, GBM, PA). The NL_UP2 spot shows slightly elevated expression in IDH-A and especially in IDH-O gliomas compared with NL_UP1.

The expression of gene sets is related to cell cycle activity [42], immune response, hypoxia and epithelial mesenchymal transition (EMT) [43] decay in IDH-mut, and especially in GBM-like tumors. Interestingly, proliferative activity concertedly decreases and immune response concertedly increases in PA and E3, at the same time systematically deviating from the expression level of these gene sets in GBM-like tumors (see arrows in Figure 2E). Parallel activation in E3 and PA was also observed for expression signatures of macrophages, activated microglia, and chemokine signaling (PA and E3 profile, Figure 2E).

PAs are virtually ‘single-pathway’ tumors arising from dysregulation of the MAPK pathways typically after lesions of the *BRAF* gene [44]. PAs (and to a lesser degree E3) show transcriptional activation of the MAPK pathway and of its targets [45], which supports the interpretation of E3 as PA-like gliomas, in agreement with [22]. In contrast, activation of the cell cycle in GBM-like gliomas (GBM_UP profile) is accompanied by low expression of E3 and PA; by low *MAPK* activity; and by upregulation of signatures of CD4+ and CD8+ T cells, together with neo-antigene signatures, all taken from [46]. These results suggest different kinds of immune infiltrations and of cytotoxicity in the tumor microenvironment (TME) in GBM-like and PA-like tumors, respectively. GBM-like (CL, MES, and to a lesser degree PN gliomas) show relative high mutational load compared with LGG, presumably causing increased generation of neoantigenes [47], which, in turn, increases tumor immunogenicity and possibly induces infiltration of CD4+ and/or CD8+ T cells, as found in GBM [48,49], LGG [24], and other cancers [50] and seen as specifically increased expression of neoantigene signatures in GBM-like tumors (Figure S4). PA-like tumors, on the other hand, show higher content of microglia/macrophages, representing intrinsic cellular constituents of the immune system of nervous tissue [51] (Figure S11). PA have been reported to exhibit marked immune response characteristics due to immune cell infiltration [52,53,54], distinctly differing from GBM [55]. Interestingly, the portrait of E2 shows a common spot appearance together with MES GMB (spot MES_UP, Figure 2D,E) assigning E2 as IDH-A gliomas with mesenchymal resemblance. Further, we compared grade IV GBM with grade II-III LGG separately in the IDH-wt and IDH-mut groups. In both cases, we found higher levels of NL_UP characteristics in LGG compared with grade IV GBM (Figure S6), which reflects the progressively decaying contribution of the NL-signature with increasing grade of the tumors in agreement with previous comparisons between grade II and III gliomas [21].

The expression SOM was transformed into a ‘prognostic map’ by linking gene activation with survival data of the respective patients. It turned out that IDH-wt signatures in the right upper corner of the map associated with high hazard ratios (HRs, colored in red) and thus with poor prognosis, while activated IDH-mut signatures associated with better outcome and upregulation of genes located in the left lower part of the map (colored in blue, Figure 2G, see also Figures S7 and S9 ). The group composition of four selected areas in the prognostic map and the respective overall survival curves illustrated the adverse effect of genes upregulated in GBM-like tumors compared with IDH-mut. Interestingly, signature genes extracted from GBM long-term survivors [56] resemble the PA and E3_UP signature in our study, which indeed shows a slightly improved prognosis compared with GBM-like tumors. Note also that the red and blue areas of high and low HR levels only partly agreed with the spot patterns of strongly overexpressed genes shown in the overexpression summary map. These differences illustrate the fact that maximum or minimum expression criteria do not necessarily associate with best or worst prognosis. Instead, subtle transcriptomic changes associate with prognosis in a more pronounced fashion with a dependence on the underlying functional context. Phenotype maps visualize the association of age and sex of the patients with their transcriptomes (Figure S8).

In summary, combined analysis of the whole transcriptome landscapes of grade I–IV gliomas including PAs revealed overlapping immunogenic signatures, especially between PA and E3, representing a PA-resembling IDH-A subtype, and between MES-GBM and E2, representing a mesenchymal-like IDH-A subtype, in contrast to E4 as a low-inflammatory IDH-A subtype. The balanced composition of IDH-wt and IDH-mut tumors in this glioma cohort provides a balanced resolution of the regulatory modes activated in both entities. Our interactive SOM-browsing tool makes the processed expression data available and enables the selection single genes, gene sets and also signaling pathway topologies, different types of phenotype maps, and information to discover their profiles across the cases and subtypes interactively (see the Materials and Methods section and [39]).

### 3.2. Cartography of the LGG Transcriptome

**Figure 3:**
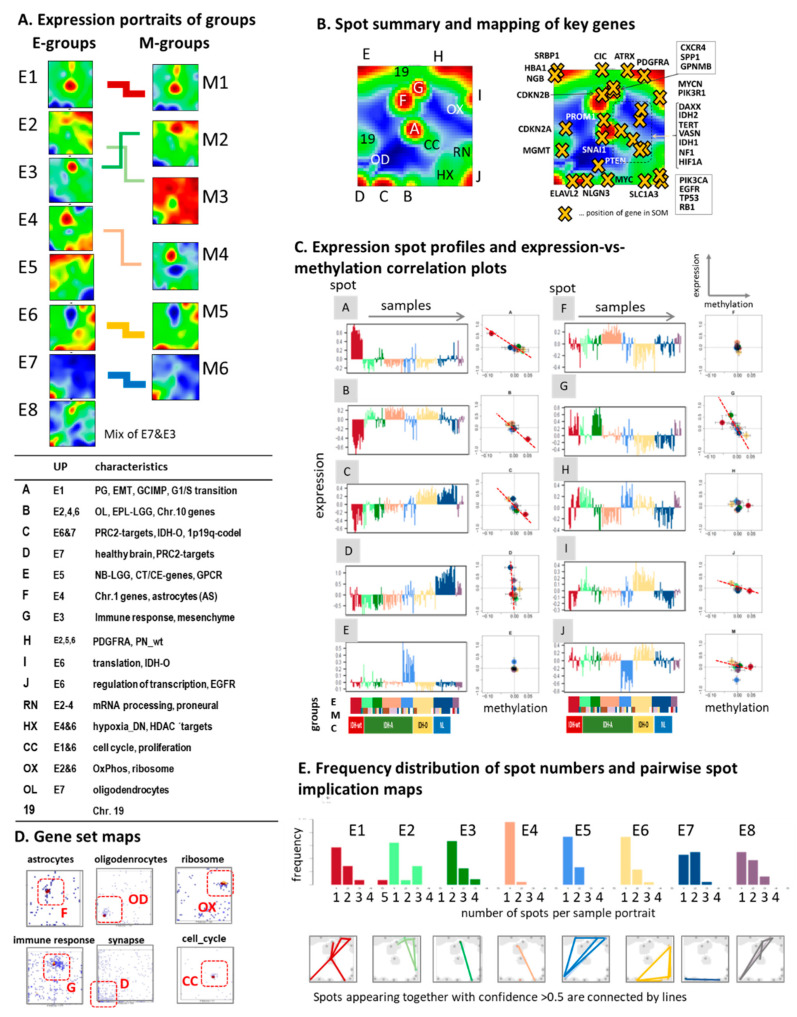
Cartography of the LGG transcriptome. (**A**) Mean SOM expression portraits of the E-groups were specific for each class and revealed correspondence with the expression portraits of the M-groups. (**B**) The LGG transcriptomes divide into 10 major modules of co-expressed genes with (**C**) characteristic profiles and mostly negative correlation between group averaged expression and gene promoter methylation (the scatter plots show mean expression versus mean methylation averaged over all genes included in the spot and all gliomas per group). (**D**) The gene set maps plot genes of selected functional context into the expression landscape. Please note their accumulation in different areas, which were assigned to the spots introduced above. (**E**) The spot-number distribution estimates the degree of the heterogeneity of sample portraits in terms of expressed spot modules and the spot implication maps join spots frequently appearing together (confidence > 0.5) in each of the subtypes by lines.

For a more detailed analysis of the transcriptomic states of LGG, we trained a ‘zoom-in’ SOM [27] that only considers LGG cases and thus enabled a closer look especially at IDH-mut tumors constituting about 85% of the LGG. All eight E(xpression) subtypes E1–E8 showed different, type-specific gene expression patterns (Figure 3A). Comparison with the expression portraits averaged over the M(ethylation) groups (see [20] and next subsection) confirmed similarities and correspondence between the E- and M-subtypes at the gene level (Figure 3A, right part). Overall, the portraits provided 10 spot clusters of co-expressed genes annotated by capital letters A to J (Figure 3B and Figure S10). Each of them showed a characteristic expression profile across the tumors that mostly negatively correlates with the mean promoter methylation levels of the genes included (Figure 3C). Their context was assigned to different functions and glioma signatures such as GCIMP genes (spot A), synaptic transmission and healthy brain (D), immune response (G), and translation and transcription (I and J, see table in Figure 3A and Table S1). Other less prominent spot regions of the portraits were associated with functions such as oxidative phosphorylation (oxphos, OX), cell cycle (CC), and hypoxia (HX) (Figure 3A,B) according to local accumulation of genes from selected gene sets (Figure 3D). 

Subtype-specific overexpression is observed for genes of spot A (GCIMP) in E1 (IDH-wt), of G (immune response) in E3 (PA-like), of F (astrocytes) in E4, of OX (oxphos) in E6 (IDH-O), and of D (synapse) in E7 (NL). Subtype E5 overexpresses spot E, which accumulates signature genes of neuroblastic LGG [58] and testis-specific genes. They are known to become activated as cancer testis (CT) genes in a wide range of cancer types [59] often encoding antigens that are immunogenic in gliomas and particularly in cancer stem cells [60,61,62]. Spot E also contains genes related to hemoglobin function such as *HBA1,* neuroglobin (*NGB*), and *SRBP1*, which upregulate in glioma tissues under hypoxic conditions [63,64,65]. Part of the spots enrich genes from different chromosomes reflecting dose–response relationships of the most prominent copy number variations in IDH-O (chromosomes 1 and 19) and IDH-wt (chromosomes 7 and 10) [20,21]. 

The distribution of spot numbers detected in the SOM portraits reveals that typically one or two activated spots were detected per portrait, which, however, combined in different ways, thus reflecting transcriptomic heterogeneity (Figure 3E). For example, E6 (IDH-O) gliomas express on average four to five spots where spots H and I were also observed in E2. E7 and E8 gliomas have slightly more spots, indicating diverse expression landscapes due to the overlap of healthy brain and glioma patterns (Figure 3E). Mutual mapping of the spot clusters identified in the LGG-SOM (Figure 3) and the ‘all-glioma’ SOM (Figure 2) revealed mostly one-to-one correspondence (Figure S10), except spots F and I, which transform into more diverse gene distributions, presumably due to different cellular compositions in IDH-wt and IDH-mut subtypes, as suggested by maps of single-cell transcriptomic signatures (Figure S11) for IDH-mut gliomas [66] and PA [67]. In summary, we identified 10 major modules of co-regulated genes that constitute the major dimensions of transcriptomic variation in LGG. They refer roughly to synaptic transmission (as a proxy for healthy brain function), proliferation (in association with cell cycle, metabolic activity, and RNA-processing), and immune response (associated with inflammation, tumor microenvironment, and stromal properties) and a clear separation between spots upregulated in IDH-A and IDH-O type tumors.

### 3.3. Cartography of the LGG Methylome

**Figure 4:**
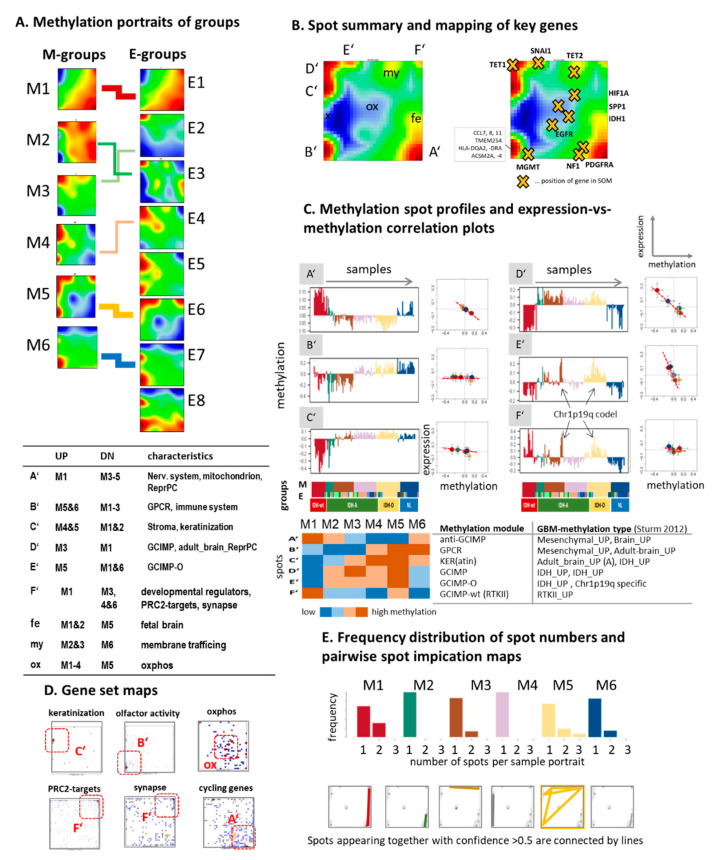
Cartography of the LGG methylome: (**A**–**E**) subgroup portraits, spot summary map, spot profiles, and functions. See legend of Figure 3 for details. The heatmap in part C provides an overview about the methylation spot modules. They were named as follows: anti-GCIMP (spot A’), resembling the CpG-HypOmethylation module of *IDH*-mutated tumors (CHOP, [29]); *GPCR*-module (B’); KER-module (C’, keratinization); GCIMP module (D’); GCIMP with specific hypermethylation of *IDH*-O (GCIMP-O, E’); and hypermethylation of *IDH*-wt, particularly of the RTK II type (GCIMP-wt, F’) [9]. The spots enrich selected GBM methylation signatures according to [9], which were taken from [29].

Next, we trained a ‘methylation’ SOM on the basis of the integral CpG methylation of the gene promoter regions. The LGG specimen group into six subtypes M1–M6 [20], revealing group-specific methylation patters (Figure 4A). They transform into similar patterns for part of the E-groups (E1, E6, E7). Slightly differing portraits of the IDH-A groups E2–E5 express the lack of clear one-to-one correspondence between the expression and methylation landscapes [20]. Overall, the obtained DNA methylation patterns are less diverse compared with the expression patterns. Only six major spot-clusters of co-methylated genes (annotated as A’–F’, Figure 4B) and mostly only one spot per sample were identified (Figure 4C). Their profiles can be assigned to a GCIMP module hypermethylated in *IDH-mut* tumors (spot D’); a GCIMP-O module with specifically enhanced methylation in IDH-O; a module hypermethylated specifically in *IDH-wt* gliomas and IDH-O (GCIMP-wt, spot F’); and an ‘anti-GCIMP’ module (A’) hypermethylated in IDH-*wt* and hypomethylated in IDH-*mut* that resemble the RTKII and the mesenchymal methylation signatures in GBM, respectively [9,20,29]. The IDH-O signature (spot F’) associates with chromosome 1p19q co-deletions. In addition, we found two hypo-methylation patterns (Figure 4C), namely, spot B’ hypo-methylated in M1–M4, which enriches genes coding G-protein coupled receptors (GPCR), and spot C’ hypomethylated in M1 and M2. Spot C’ enriches genes related to epidermal cell differentiation and keratinization, which are prone to hypo-methylation also in other cancers [68]; tune the balance between stemness and somatic functions [69]; and promote epithelial–mesenchymal transition EMT-like processes [70,71]. Clear negative correlations between methylation and expression were found only for three modules (A’, D’, and E’), while the other three showed virtually no change of the mean expression with methylation. Moreover, only a few characteristics of the methylation spot modules such as the enrichment of *PRC2* targets in the GCIMP-wt module reproduce the functional context of the expression spots, which is in line with the lack of one-to-one correspondence between the methylation and expression landscapes established above for the IDH-A groups and previously also for GBM [28] and differentiating cell lines [72]. In summary, the LGG methylome decomposes into six major modules of co-methylated genes, which besides GCIMP associates with the ‘olfactory’ subgenome [20,73] and inflammation, keratinization, and EMT. Note also that we identified the methylation of the olfactory subgenome as the main factor governing a subtle ‘resorting’ of tumors between E- and M- groups (spot B’, Figures S13 and S14). It not uniquely associates with the expression and/or chromosome 1p19q co-deletion state of the tumors leading to slightly modified class assignments.

### 3.4. Glioma Key Genes Support the Functional Impact of the Molecular Maps

The functional context of different regions of the expression and methylation maps is further supported by the location of glioma key genes within or near the spots, which indicates co-regulation. For example, the genes *IDH1/2*, *DAXX*, *TERT*, *NF1*, and *HIF1A* co-express with the cell cycle and oxphos signatures (Figure 3B). The receptor tyrosine kinases (RTK) *EGFR* (Chr7) and *PDGFRA* (Chr4) are genetic drivers of gliomagenesis [8,9,36,74,75]. Overexpression of *EGFR* (spot J) in E4 corresponds to frequent gains on chromosome 7 in this subtype [20]. Genetic lesions of *TP53* and *ATRX* are specific for astrocytic tumors [76], however, with activating and de-activating consequences on expression. In consequence, *TP53* co-regulates with *EGFR* (spot J, up in E4 and E6) together with the glutamate asparagate transporter *SLC1A3*, a marker for early stage neuroglial progenitors [58], while *ATRX* downregulates in IDH-A. The oligodendroglial marker *CIC* (Chr19) locates in an area of the map enriching genes from chromosome 19 [66,77,78,79,80]. *ELAVL2*, a marker for differentiated migrating neuroblasts [58], co-expresses with spot C upregulated in IDH-O (E6) and NL (E7) tumors together with the synaptic adhesion molecule neuroligin-3 (*NLGN3*) promoting proliferation [81]. Activated proliferation in LGG and adverse prognosis associate with downregulation of the tumor suppressor *CDKN2A/B* located near spot F [82,83]. The marker genes for macrophage expression in glioma *SPP1* and *GPMB* [84] co-regulate with spot G enriching also other genes with impact for inflammation, among them *CXCR4*, related to glioma associated angiogenesis [85]. The stemness marker *PROM1* (*CD133*) and the driver of the epithelial–mesenchymal transition *SNAI1* [86] are found near spot A, which collects GCIMP genes mostly underexpressed in *IDH-mut* tumors. Interestingly, most of the ‘key’ genes discussed are only weakly affected by promoter methylation (Figure S15), meaning that they are preferentially regulated by genetic lesions and/or transcription factor networks and to a lesser degree by epigenetics [28].

The *GPCR*-cluster (spot B’, Figure 4B) contains genes coding chemokine ligands such as *CCL-7, -8*, and *-11* and also human leukocyte antigens (*HLA*) such as *HLA-DRA2* and *-DQA*, all related to immune response. De-methylation of *GPCR*, especially in M1-M3, associates with the activation of immune response, which suggests functional association. The gene promoters of *TET1* and partly also of *TET2* encoding DNA demethylases methylate in parallel with the GCIMP and GCIMP-wt methylation patterns, respectively. These changes suggest negative feedback mechanisms between methylation and expression and thus amplification of hyper-methylation patterns via suppression of DNA de-methylation in addition to their metabolic repression via 2-HG in *IDH*-mut tumors [87]. Hence, SOM-cartography of genes with key functional impact for glioma pathophysiology provides a consistent picture with previous knowledge and links them with regulatory gene modules of concerted expression and/or methylation.

### 3.5. Epi-Genome and -Transcriptome Modifiers, Telomere Maintenance, and Single-Cell Signatures

In addition to single genes and sets of central cellular functions such as cell cycle (Figure 3D) we mapped sets of genes with potential impact for glioma biology into the molecular landscapes, namely, genes coding epi-genome (Figure S16) and -transcriptome (Figure S17) modifying enzymes (writers, readers, and erasers of methylation marks at DNA, RNA, and histones), genes with impact for telomere maintenance by means of telomerase or alternative lengthening mechanisms (Figure S18), and cell-related gene signatures derived from single-cell transcriptome studies on gliomas (Figure S11). Epigenome-related genes upregulate specifically in IDH-mut subtypes as indicated by their accumulation in/near spot H (E2) and B (E6) (Figure S16) thus reflecting subtle de-regulation of the epigenetic machinery between the subtypes with possible consequences for DNA methylation. Epitranscriptome-related genes are activated in gliomas compared with brain-like NL tumors. Interestingly, they resemble the activation patterns of ‘canonical’ splicing gene sets (Figure S17), which underpin similar functional impact in modifying RNA processing. Expression of a series of DNA- (e.g., *DNMT1*) and histone-methyltransferases correlates with cell cycle-activating gene sets presumably to ensure maintenance methylation at DNA and histones in highly proliferating cells. In contrast, expression of a series of RNA-methyltransferases and -demethylases correlates with genes of ribosomal and oxphos functions. Interestingly, genes of epi-transcriptome and -genome functions deplete in spots A (GCIMP), D (synapse, *PRC2* targets), F (astrocytes), and G (inflammation), paralleled by anti-GCIMP methylation characteristics, reflecting their overall impact in gliomas.

Telomere maintenance (TM) is another mechanism governing glioma biology in a subtle way [88]. Almost mutually exclusive mutations of the *TERT* gene promoter in IDH-wt and IDH-O tumors on one hand, and of the *ATRX* gene body often in combination with mutated *TP53* in IDH-A gliomas on the other hand give rise to TEL(omerase induced) and ALT(ernative)-like TM pathways, respectively [22,89]. Genes of these pathways distribute in characteristic patterns in the expression landscape, indicating activation of *TEL*-related genes in IDH-wt and IDH-O tumors and deactivation of *ATRX* in IDH-A gliomas (Figure S18).

As a final category, we mapped cell type-specific expression signatures taken from recent single cell RNAseq studies (Figure S11). They clearly assign spots F (to astrocytes and astrocytoma cells), C (to oligodendrocytes and oligodendroglioma cells), and G (to microglia/macrophages) to the respective cell types. In summary, maps of genes with different functional impact resolve the topology of the molecular maps beyond the spots of highly expressed (and/or methylated) genes. These details associate with a fine tuning of gene activity as a result of a subtle interplay between aberrant methylation in concert with reshaped epi-genome and -transcriptome, telomere maintenance, and the tumor cell microenvironment.

### 3.6. Glioma Progression and Recurrence

**Figure 5:**
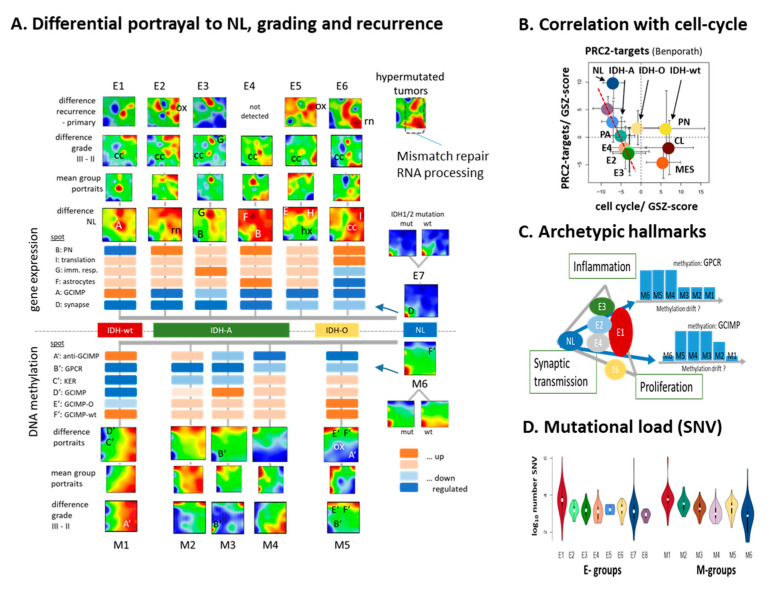
Alterations of transcriptional programs and of DNA methylation patterns between subtypes and upon recurrence: (**A**) differential SOM-portraits of the E- and M-subgroups with respect to the NL subtype revealed alterations of expression and methylation patterns upon glioma development and their functional context. Stratification of portraits of E7 and M6 with respect to the *IDH* mutation status showed almost identical patterns, meaning that E7 and M6 are suited as reference state, which is dominated by healthy brain characteristics. Differential portraits were also calculated between WHO grades III and II (see also Figure S19) and between primary and recurrent tumors [24]. Cell cycle (CC), oxphos (OX), and/or inflammatory (G) spots gain in most comparisons. (**B**) Group-averaged expression (GSZ-score) of PRC2 targets as a function of cell cycle activity. Overall, PRC2 targets negatively correlate with cell cycle where effect is largest for IDH-wt (CL and MES subtypes). For IDH-A tumors, one finds a nearly linear decay as indicated by the red line. (**C**) The summary scheme locates the subtypes in triangular coordinates spanned by ‘archetypic’ cellular functions. It shows selected methylation profiles indicating reduced levels in of the IDH-A subtypes. (**D**) Mutational load (log number of single nucleotide polymorphisms) per tumor is largest in IDH-wt (E1, M1) and smallest in NL (E7, E8, M1). It increases upon IDH-A progression in the order M4, M3, M2. SNV numbers were taken from TCGA-matched LGG [20].

Next, we characterized systematic changes of the expression and methylation patterns of gliomas compared with neural subtypes E7 and M6, respectively. They virtually resemble healthy brain characteristics due to reduced tumor cell content, and therefore we used them as internal reference to describe tumor development ([20], Figure S6). The difference maps in Figure 5A (see also Figure S12) reveal consistent downregulation of healthy brain functions (spot D), upregulation of cell cycle, and translational and oxphos activities (spots I and ‘OX’) in all glioma subtypes compared with NL. Immune response was increased, especially in E3 and also in the other IDH-A subgroups, but decreased in IDH-O (E6, spot G). These expression changes are paralleled by consistent hyper-methylation of functions related to synaptic transmission associated with de-repression of *PRC2* targets and by hypo-methylation of the oxphos signature, specifically in M5 (IDH-O). The methylation gene signature is related to *GPCR* and keratinization loose methylation in all glioma subtypes compared with M6; however, the strongest effect was observed in M1–M3. The plot of *PRC2* target expression versus cell cycle activity confirms their virtually antagonistic relationship seen in the portraits (Figure 5B). 

For a schematic overview, we distributed the different subtypes in a triangular coordinate system with the cellular functions inflammation, proliferation, and synaptic transmission (healthy brain function) as ‘archeotypic’ glioma tasks along the coordinates (Figure 5C, [90]). Accordingly, all gliomsa except E7 loose synaptic function; IDH-O (E6) showed the lowest inflammation, but relatively high proliferative activity, while IDH-wt combines high proliferation with inflammation. The IDH-A subtypes activity indicate increasing inflammatory characteristics along the E4–E2–E3 axis. Major methylation profiles across subtypes showed hypermethylation (GCIMP and GCIMP-O) and/or hypomethylation of the *GPCR* subgenome. Interestingly, the respective methylation levels differ between the IDH-A subtypes, e.g., *GPCR* methylation dropped markedly in M2 and M3 compared with M4 while M2. These methylation changes possibly reflect dynamic alterations of the DNA methylome, so-called ‘methylation’ drifts also observed upon ageing of healthy cells. They relate to cellular programs and presumably the ‘age’ of the tumor. For example, incomplete maintenance methylation in high proliferation glioma cells is expected to reduce methylation upon tumor development accompanied by ‘drifts’ of tumor methylation and transcriptional state. For an independent evaluation of the tumor age, we counted the mutational load as the number of single nucleotide polymorphisms (SNVs) per gliomas (Figure 5D). IDH-wt showed the largest value, while mutational load increased along the IDH-A groups M4–M3–M2 in parallel with the loss of overall methylation [20] and methylation of the *GPCR* (B’) and keratin (C’) modules (Figure 4C).

Stratification of the subtype portraits into grade II and III tumors indicates that the differential spot signatures with respect to NL tumors gain in higher grade III tumors compared with grade II tumors, meaning that of subtype-specific characteristics diverge upon tumor progression (Figure S19). We also included independent longitudinal follow-up RNAseq data taken from [24], generated their SOM portraits, stratified them according to our subtypes, and subsequently calculated difference portraits between recurrent and primary gliomas (Figure 5A, row above). Interestingly, the difference between recurrent and primary tumors resembles the difference portraits between grade III and II tumors, meaning that recurrence molecularly follows a similar trend as increased WHO grade. 

Overall, these results show that tumor development in a direction of increasing WHO grade and/or after recurrence ‘intensifies’ cell cycle and metabolic activities in virtually all subtypes and inflammatory cellular programs, specifically in E3. On the methylation side, these trends are paralleled by subtle losses (e.g., OX and A’ in M5 and also GCIMP/D’ in M2–M5) and/or gains (GCIMP-O in M5) of methylation modules, suggesting that changes of cell activities are partly driven by hypo- and/or hypermethylation drifts of the promoters of associated genes. Recurrence is accompanied with hypermutation bursts in part of the tumors [24]. Their mean expression portrait shows patterns resembling activated DNA mismatch repair (Figure 5A, right part), RNA-processing (Figure S17), and telomere maintenance (Figure S18), all associated with increased proliferation, transcription, translation, and telomere maintenance. In summary, glioma development with increasing grade and upon recurrence associates with increasing cell cycle and immune response (E1–E3) and decaying *PRC2* target and healthy brain activities (all subtypes) presumably driven by methylation drifts.

### 3.7. (De)coupled Alterations of Expression and Methylation Patterns 

**Figure 6:**
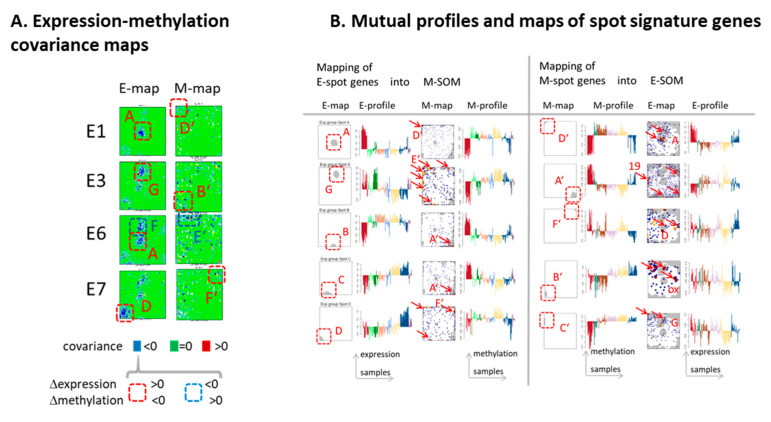
Relations between expression and methylation: (**A**) The covariance maps highlight genes co-regulated by mostly anti-correlated expression and methylation values. The frames mark selected spot areas where red and blue color assign opposite alterations of expression and methylation levels as indicated in the figure. (**B**) Mutual mapping of E- and M-spot genes (red frames) into the M- and E-SOM (arrows) indicates spot ‘melting’ and suggests divergence of genes related to distinct molecular mechanisms with respect to concerted transcriptional activation and methylation changes. Note that capital letters in the SOM-portraits assign spot modules.

In order to specify possible co-regulation of gene expression and -methylation, we generated co-variance maps of selected subtypes (Figure 6A). These maps color code the values of covariance calculated between the expression and methylation of the genes collected in each pixel of the maps. The E(xpression) covariance maps revealed marked anti-correlations between the expression and methylation levels in regions assigned to GCIMP genes (spot A), immune response (G), the astrocyte signature (F), and synaptic transmission (D) (Figure 6A, left part). The co-variance characteristics were less pronounced and fuzzier in the M(ethylation) maps, which suggests decoupling, especially between co-methylated genes and their mean expression values (Figure 6A, right part). To better understand this decoupling effect, we mapped genes from selected spot clusters of the E-map into the M-map and vice versa (Figure 6B). Genes taken from spot A (GCIMP) accumulated relatively uniquely into spot D’ in the M-map, while spot D (brain) split into three spots (B’, D’, F’; referring to *GPCR*, GCIMP, and *PRC2* targets, respectively), thus reflecting three different methylation patterns of genes related to healthy brain function. Spot G (immune response) in the E-map ‘melted’, meaning that the spot genes distributed over larger areas of the M-map, which indicated the lack of unique methylation effects. The inverse mapping of spot genes from the M-map into E-map showed a similar spot ‘melting’, and thus the absence of a one-to-one relationship between co-expression and co-methylation. In other words, transcriptional programs are often affected by aberrant methylation of only part of the involved genes and vice versa, and co-methylation can affect different transcriptional programs. This asymmetry can be rationalized by the fact that gene activity is governed by different factors such as genetic lesions, alterations of the chromatin state, and/or interactive networks of transcription factors [72], which can act independent of DNA methylation and form ‘hidden’ regulatory layers partly decoupling gene expression and methylation. 

### 3.8. A Combined Network of Transcriptional and of Methylation Modules

**Figure 7:**
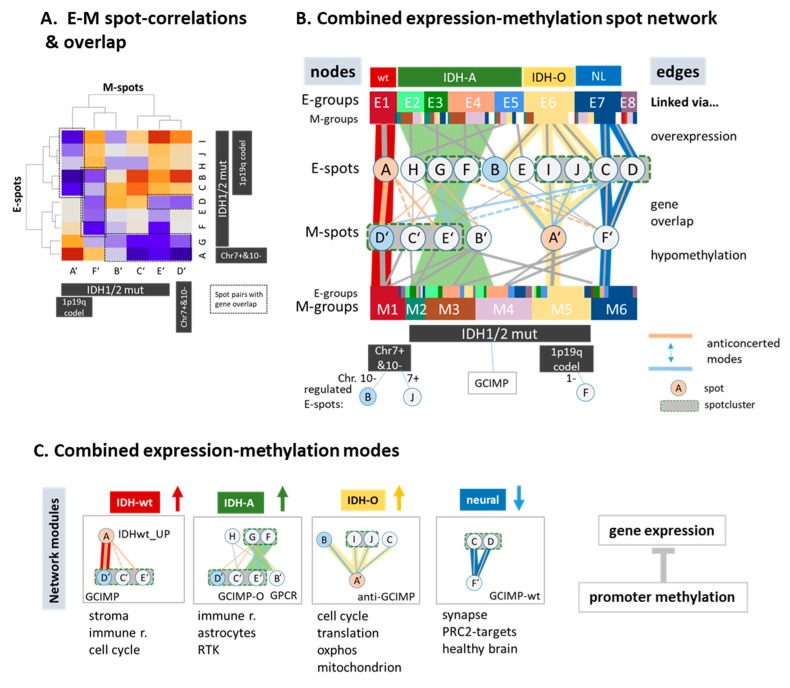
Combined view of the expression and methylation landscapes: (**A**) The network between expression and methylation spots (see Figure S20 for details) was obtained on the basis of gene overlap between the spots and mutual correlations between the spot profiles. (**B**) The correlation map between expression and methylation spot values revealed negatively correlated M–E spot pairs with considerable gene overlap (see also Figure S20). (**C**) The network in part A divided into four main coupled regulatory modules in the four consensus subtypes.

For an integrative view on mutually associated expression and methylation changes, we correlated all spot clusters in the E- and the M-map, selected those with considerable gene overlap (*p* < 10^−3^, Fishers test; Figure 7A and Figure S20), and constructed a network of anti-correlated expression and methylation spots (Figure 7B). The net split into four major combined regulatory modes that associate with the four consensus subtypes, reflecting modes of genomic regulation affected by gene promoter methylation (Figure 7C). The neuronal (NL) and the oligodendroglial (IDH-O) subtypes were governed by spots F’ and A’ on the methylation side, with impact for synaptic transmission and energy metabolism, respectively. On expression side, gene activity split into more than one spot per module due to the higher diversity of expression patterns. For IDH-wt tumors, this relation reversed, meaning that one expression spot A co-regulated virtually with three methylation spots. They all included GCIMP genes not suppressed in IDH-wt gliomas and diversified the different IDH-A subtypes into immune response (especially E3), astrocyte (especially E4), and RTKII/MES (E2) signatures. In summary, our network analysis extracted four major modules coupling gene expression and promoter methylation. They associated with activated transcriptional programs in IDH-wt, IDH-A, and IDH-O type gliomas and deactivated healthy brain function in all subtypes. 

### 3.9. Phenotype Maps: Prognosis, Age, and Telomere Length

**Figure 8:**
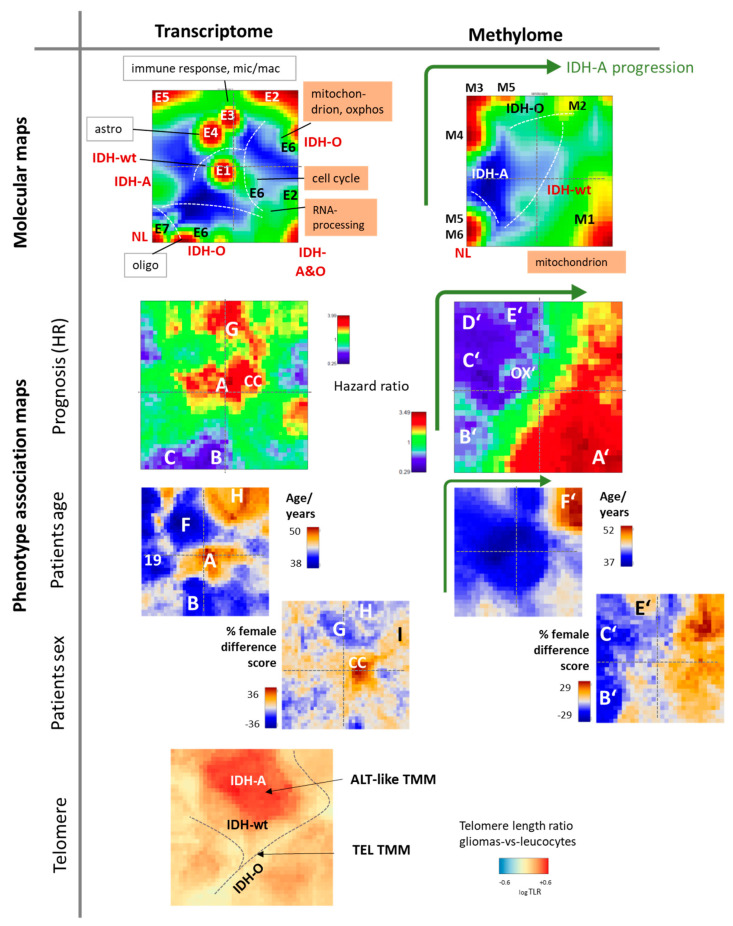
Molecular and phenotype maps of the transcriptome (left part of the figure) and methylome (right part) landscapes of LGG. Phenotype maps show associations between gene expression and promoter methylation with the hazard ratio (HR), patient’s age at first diagnosis, sex of the patients, and telomere length ratio between tumor and leukocytes (TLR). Phenotype maps were generated as described in Figure S7 for prognostic maps. The ‘female difference score’ estimates the deviation from the mean percentage of female patients in units of ‘percent-of-percent’. Overall, phenotype maps enable the comparison of expression and methylation levels in the different subtypes with the respective phenotype features.

Expression and methylation data landscapes link overexpression and hypermethylation with cellular programs activated in different cell types, glioma groups, and stages of tumor development (see ‘molecular maps’ in Figure 8 first row). ‘Phenotype’-association maps relate the molecular expression and methylation maps to patient’s properties such as overall survival hazard ratio (HR), age, and sex (Figure 8, second row). The methylome prognostic map divided into two major areas of high and low HR, referring to genes either hypermethylated in *IDH*-wt tumors or in *IDH-mut* ones [21]. Low HR and thus good prognosis was associated with enhanced *GPCR* methylation (spot B’) in NL (M6), IDH-O (M5), and also in M4 tumors, which enrich lower-grade II IDH-A gliomas. Moreover, the keratin (spot C’) and GCIMP (D’) modules were associated with good prognosis where methylation decayed along these spots and subtypes in direction M4–M3–M2, suggesting association with tumor progression as indicated by the green arrow in Figure 8 (see also Figure S12B). The prognostic transcriptome map was more structured than the methylation map. Poor prognosis was associated with ‘overexpression’ of GCIMP genes in *IDH*-wt tumors (spot A) and also with inflammation mostly in E3 IDH-A type gliomas (spot G). Blue areas of better prognosis were indicative for E7 (neural gliomas), partly E4 (mostly grade II astrocytomas) and E6 (IDH-O). Elderly patients and slightly higher percentages of women were associated with areas of worse prognosis (spots A and CC). Among IDH-A gliomas, E4 (enriched in grade II, spot F) refers more to younger patients while E2 (spot H, grade III enriched) and also E3 (spot G, inflammation, microglia, macrophages) to more to elderly ones (see also Figure S19). Sexual dimorphism arose also between spot G and CC (cell cycle), which eventually supported the view that such differences act at the level of tumor microenvironment and, particularly, glioma-associated macrophages ([91] and references cited therein).

Association with telomere length indicated relative large values in an area of IDH-A overexpression but relatively low values in a region of combined overexpression of IDH-O and IDH-wt (Figure 8 and, for details, Figure S18 and [22,89]). This picture is in agreement with the fact that majority of IDH-A activate an ALT-like telomere maintenance mechanism after mutation of *ATRX*, which gives rise to longer nominal telomere lengths [22,89]. In contrast, most IDH-O and a large fraction of IDH-wt gliomas maintained telomere lengths via activation of telomerase (TEL) after promoter mutations of the *TERT* gene, which kept telomere lengths short but slightly above the telomere crisis threshold. Interestingly, ALT mechanism was associated with immunogenic IDH-A phenotypes of varying levels of methylation, while TEL was found in highly proliferative IDH-O and IDH-wt gliomas. Telomere maintenance via TEL mechanisms is typically found in highly proliferating tumors of epithelial phenotypes such as high-grade melanomas and colon cancer, while ALT is more prone to mesenchymal immunogenic tumors such as sarcomas undergoing epigenetic reprogramming [92,93,94]. Hence, SOM visualization of molecular and cellular properties is complemented by information about disease-related phenotypic features, which enables identification of associations with molecular functions and potential marker genes.

## 4. Discussion

### 4.1. Combinations of Proliferative, Inflammatory, and Tissue Remodelling Functions Shape Glioma Phenotypes

**Figure 9:**
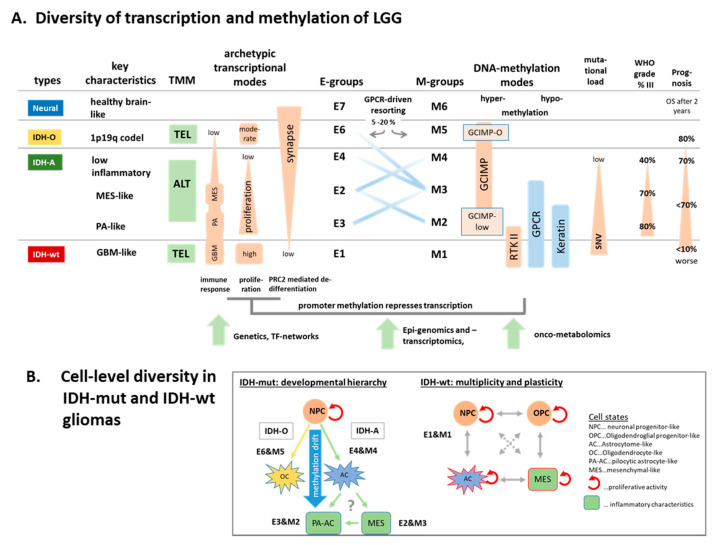
Schematic overview: (**A**) Major characteristics, cell functions, and methylation modes of the LGG types. (**B**) Cell-level view suggesting that aberrant DNA methylation shapes IDH mut gliomas into a developmental hierarchy while IDH-wt gliomas consist of multiple cellular states. The scheme was partly adapted from [98] on the basis of recent single-cell transcriptomic studies [66,67,99,100] and our mapping of single cell glioma signatures (Figure S11). See text.

Molecular subtyping has emerged as an important concept to decipher cancer heterogeneity and to better understand the underlying biology. Our ‘all glioma’ analysis combined GBM, PA, and LGG in a cohort of a balanced numbers of tumors with and without the IDH1/2 mutation. It trivially reflects the massive effect of the IDH1/2 mutation on the whole transcriptome landscape [22] and prognosis [21] of the tumors. Increasing WHO grade from grade II LGG to grade IV GBM progressively suppressed healthy molecular brain functions driven by epigenetic de-differentiation mechanisms (see Figure 9A for an overview). The IDH-mut transcriptome split into an IDH-O (oligodendroglioma-like, E6) and IDH-A (astrocytoma-like, E4–E2) gene expression pattern. IDH-A associated with inflammatory characteristics partly resembling signatures observed in GBM mesenchymal (E2 IDH-A) and PA tumors (E3). Inflammatory characteristics are low in IDH-O, but progressively increase in IDH-A in the order E4–E3–E2 which associate with senescence and treatment resistance characteristics, decaying GCIMP-methylation and worse prognosis [20]. Recent studies developed risk scores related to DNA methylation and inflammatory tumor microenviroment resembling our characteristics [95,96]. 

The transcriptional patterns of LGG can be summarized into three ‘archetypic’ hallmarks referring to increased proliferation, inflammation, and loss of original brain tissue function. Accordingly, IDH-wt (E1 group) combines, first of all, inflammatory and proliferative characteristics while IDH-O combines moderate proliferative and partly healthy brain functions. IDH-A tumors are more ‘generalists’ by combining all three glioma tasks on intermediate levels and with stronger inflammatory PA-like contributions in E3 and metabolic and transcriptional activities in E2. Note that our evaluation of proliferative activity is based on expression of gene signatures of cell cycle activity [42], which have been shown to be associated with histochemical assays such as KI67 in other cancer entities [31]. In gliomas, high KI67-values are associated with inferior prognosis, while the method and other assays such as mitotic counts is still under discussion, particularly when comparing different glioma entities [97]. IDH-O gliomas have overall slightly better prognosis than IDH-A [21], despite their elevated cell cycle activity, possibly because of its composite character in bulk transcriptomics originating from different cell types and also other factors such as tumor microenvironment, eventually affecting prognosis.

We identified about one dozen expression modules of co-regulated genes of different functional context arising in (higher) grade IV and (lower) grade II-III gliomas as well. Analysis of the glioma methylome revealed a less diverse landscape of six subtypes M1–M6. Hypermethylation was observed first of all in GCIMP-related genes of IDH-mut gliomas, while hypomethylation was associated predominantly with the olfactory subgenome in IDH-wt in part of the IDH-A gliomas (M1–M3). Negative correlation between promoter methylation and gene expression was the dominating interaction mode, giving rise to four major network motifs of coupled gene expression and promoter methylation in terms of activated cellular programs in IDH-wt-, IDH-A-, and IDH-O-type gliomas and the deactivation of healthy brain function in all subtypes. Partial decoupling between transcription and methylation on the level of regulatory modes can be rationalized by ‘hidden layers’ of genetically (mutations and chromosomal aberrations), epigenetically, and transcription factor-driven mechanism [72]. 

### 4.2. Glioma Progression, Immuno-Ageing, and Transitory States

Hypermethylation due to the IDH1/2 mutation is caused by metabolic repression of enzymes erasing DNA and histone methylation. Resulting DNA hyper-methylation de-differentiates neuronal tissue, this way creating a glioma phenotype by epigenetic re-modelling [101]. This GCIMP mechanism is modulated in IDH-O gliomas, giving rise to a specific GCIMP-O hypermethylation signature that is associated with activated proliferation compared with IDH-A, which is, however, smaller than in IDH-wt gliomas. The GCIMP signature showed reduced methylation levels in IDH-A tumors of the M2-type similar to the GCIMP-low gliomas reported previously [22]. It partly resembled the RTKII methylation state observed in grade IV GBM [9,20,28] and makes M2 an intermediate molecular subtype linking IDH-wt and IDH-mut tumors. On the expression side, M2 corresponds to E3, partly resembling IDH-wt immunogenic characteristics. Hence, IDH-mut gliomas seem to pursue either proliferative or immuno-editing survival strategies for IDH-O and IDH-A, respectively. Interestingly, these characteristics associate with telomerase-driven or alternative telomere maintenance mechanisms, respectively, which, in turn, agrees with the preference of TEL and ALT mechanisms for tumors of epithelial or mesenchymal phenotypes, respectively. Previous pan-cancer analyses suggest that GCIMP stands out from high methylator (CIMP-H) non-glioma tumor entities such as colon cancer by displaying lack of proliferation [102]. Such conclusions might be misleading without stratification of IDH-mut gliomas into subtypes, particularly into IDH-O and IDH-A, strongly differing in proliferative and inflammatory characteristics.

Hypomethylation in gliomas splits into two major patterns, which enrich genes of the olfactory sub-genome coding a series of G-protein coupled receptors (*GPCR*) and/or genes related to epidermal cell differentiation and keratinization, which are prone to hypo-methylation also in other cancers [68]. The affected genes loose methylation in IDH-wt, in part of the IDH-A and only in a few IDH-O (chromosome 1p19q codel) tumors. The loss of methylation of the olfactory sub-genome from M6 to M1 possibly reflects a global hypomethylation drift, presumably due to incomplete re-constitution of methylation after each cell division and, in consequence, accumulation of methylation loss with tumor age. This view is supported by the increasing WHO grade, mutational load, and global hypomethylation observed in the IDH-A tumors along the M4–M3–M2 axis. Recent results indeed report a delay in maintenance methylation during accelerated cell division in gliomas [11]. 

Hypomethylation is observed with development of many cancer types in certain analogy with ageing of healthy tissues where detailed mechanisms are still unclear. The role of DNA methylation as a molecular link between aging and cancer seems more complex [101,103,104]. On the other hand, ageing of healthy tissues often accompanies increasing inflammation (‘immunoageing’) and senescence at the cell level [105]. Senescence signatures also show increasing levels in IDH-A gliomas along the M4–M3–M2 and E4–E2–E3 axes of tumor age in parallel with increasing scores of treatment resistance and progression [20,95], suggesting that the immune microenvironment shapes the evolutionary trajectories of IDH-A gliomas by generating different immunogenic subtypes [24]. De-methylation of the *GPCR* (spot B’) and keratin (spot C’) signatures associate with activation of inflammation and epithelial–mesenchymal transition EMT-like-processes- [70,71]. Note that *GPCR* are involved in aging and senescence of neural tissues via degradation of cellular signaling [106] and in differentiation of neuronal tissues [107]. Overall, the demethylation of the olfactory subgenome in gliomas seems to associate with tumor age, whereas the possible consequences need further studies. 

### 4.3. Archetypic Hallmarks, Developmental Hierarchy, and Plasticity

The dynamic and reversible nature of DNA methylation may facilitate phenotypic plasticity by modulating the archeotypic hallmarks driving glioma heterogeneity. Tumor evolution via natural selection and adaptation might act not only upon stable heritable genetic alterations, but may also operate through non-genetically encoded epigenetic states [108]. Glioma cell populations consequently may face a trade-off between different tasks where a cell that is ‘best’ at a certain task (for instance proliferation) represents a specialized archetype, e.g., in IDH-O, possessing distinct methylation and expression properties. Phenotypes that lie between specialized archetypes, such as inflammation and proliferation, in gene expression and/or methylation space are more ‘generalists’ such as IDH-A that have better survival chances in a changing immunogenic microenvironment. 

Recent single cell transcriptomics studies added new knowledge that supports this view (Figure 9B, [98]). Accordingly, IDH-mut gliomas are characterized by a developmental hierarchy constituted of cells resembling neuronal progenitors (NPC) and two subpopulations of differentiated glia-like cells resembling oligodendrocytes (OC) and astrocytes (AC). Proliferation of NPCs drives tumor growth into the separate IDH-O and IDH-A branches, mainly due to the different genetics (e.g., chromosome 1p19q co^-deleted versus intact features). Our results suggest development along the IDH-A branch via E4 towards E2 and E3 transcriptomic states with correspondence to the methylation states M4, M3, and M2, respectively. In contrast, IDH-wt are assumed to consist of four malignant cellular states; three neurodevelopmental ones related to NPC, AC, and oligodendroglial progenitors (OPC); and a fourth mesenchymal (MES) cell type, which all are proliferative and of high plasticity, meaning that they are able to transform into each other. Different IDH-wt subtypes constitute mixtures of these four cell states that combine in different proportions in dependence of genetic, epigenetic, and microenvironmental factors. Our results suggest that changing methylation in IDH-mut gliomas serves as the main factor that drifts them along the two branches where the IDH-A states develop into MES- and PA-like AC states, e.g., due to immune-aging, as discussed above.

## 5. Conclusions

Our study demonstrates the diversity of molecular patterns of LGG by stratifying gliomas into subtypes and regulatory modes governed by combinations of genetic, epigenetic, and transcriptomic factors. Astrocytoma-like IDH-A gliomas associate with different types and levels of immune cell infiltration and DNA methylation characteristics, making them different from more proliferating IDH-O gliomas. With GCIMP low methylation and PA-like transcriptional characteristics, IDH-A creates a link to GBM. Personalized, case-by-case views are necessary to interpret IDH-A gliomas as a continuous entity with respect to molecular heterogeneity and development. Hereby, SOM cartography can serve as a graphical coordinate system that links gene-level information including key genes and co-regulated ‘signature’ gene sets with glioma subtypes and their functional context. Overall, these results further support the importance of epigenetics for glioma diversity and development, and thus also for epigenetic-directed treatment [109]. 

## Data Availability

Results of transcriptome and methylome analyses presented in this publication can be interactively discovered regarding further details using the oposSOM browser [39] available in the internet via the IZBI-portal (https://www.izbi.uni-leipzig.de/opossom-browser/) or under https://apps.health-atlas.de/opossom-browser/?dataset=3 (LGG expression dataset), https://apps.health-atlas.de/opossom-browser/?dataset=9 (LGG methylation dataset), and https://apps.health-atlas.de/opossom-browser/?dataset=4 (all grade glioma dataset). Gene expression and methylation data are available in the gene expression omnibus (GEO) database under accession number GSE61374 (LGG expression [21]), GSE129477 (LGG methylation [20]), and GSE53733 (GBM expression [23]).

## Supplementary Materials

The following are available online at https://www.mdpi.com/article/10.3390/cancers13133198/s1, Supplementary File 1: Figure S1: Functional analysis using the functional category ‘Gene Ontology Biological Process’ (GO BP). Figure S2: Functional analysis using the functional category ‘Gene Ontology Cellular Component’ (GO CC). Figure S3: Functional analysis using gene sets of the functional category ‘hallmarks of cancer’. Figure S4: Functional analysis using gene sets of the functional category ‘immunome’. Figure S5: Pairwise differences between the SOM expression portraits of glioma. Figure S6: Comparison of selected expression gene set profiles between grade IV (GBM) with grade II-III (LGG) gliomas. Figure S7: Prognostic maps. Figure S8: Phenotype maps. Figure S9: Overall survival (OS) curves of the glioma groups studied. Figure S10: Mutual mapping of the spot clusters between the ‘all-gliomas’ and ‘LGG-only’ SOMs. Figure S11: Mapping of single-cell glioma signatures extracted from IDH-mut gliomas and pilocytic astrocytomas (PA) into the ‘all-glioma’ and LGG SOM. Figure S12: Pairwise difference SOM portraits between the mean portraits of the expression (part A) and methylation (part B) subtypes. Figure S13: Stratification of the E- and M-subtype portraits into mean portraits of chromosome 1p19q-codeleted and -intact tumors. Figure S14: Resorting of tumors between E- and M-groups owing to different methylation of the olfactory subgenome. Figure S15: Selected glioma key genes and the correlation between their expression and DNA methylation levels. Figure S16: Gene expression and DNA promoter methylation of chromatin-modifying enzymes. Figure S17: Expression of genes coding epi-transcriptome-modifying enzymes. Figure S18: Telomere maintenance in gliomas. Figure S19: Stratification of the E- and M-subtype portraits into mean portraits of WHO grade II and III tumors and of the difference between them. Figure S20: Construction of the mutual network between expression and methylation spots. Table S1: Functional enrichment of expression spots. Table S2: Functional enrichment of methylation spots. Table S3: Gene lists of expression spot clusters A–J; Excel Table. Table S4: Gene lists of methylation spot clusters A’–F’; Excel Table.

## Abbreviations

2HG	2-hydroxyglutarate
ALT	alternative lengthening of telomerse
BMBF	Federal Ministry of Education and Research
BP	biological process
C1–C4	consensus classes of LGG with mutual correspondence between expression and methylation: C1- IDH-wt; C2—IDH-A; C3—IDH-O; C4—NL
CC	cell cycle
CL	classical subtype of grade IV gliomas according to Verhaak et al. (2010)
CHOP	CpG-hypomethylation pattern of IDH-mutated tumors
CN	copy number
E1–E8	expression subtypes of LGG
EMT	epithelial–mesenchymal transition
EPL	early progenitor-like subtype
GBM	glioblastoma WHO grade IV
GCIMP	glioma CpG-island hypermethylation phenotype
GCIMP-O	GCIMP with specific hypermethylation of IDH-O
GCIMP-wt	hypermethylation of IDH-wt, particularly of the RTK II type
GEO	gene expression omnibus database
GGN	German Glioma Network
GPCR	G-protein-coupled receptor
GSZ	gene set Z-score
HLA	human leukocyte antigen
HM	hallmarks of cancer
HR	hazard ratio
hx	hypoxia
IDH	isocitrate dehydrogenase
IDH-A	malignant astrocyte-like, IDH-mutated with chromosome 1p19q intact
IDH-mut-codel	IDH mutant additionally carrying co-deletion of chromosome arm 1p and 19q
IDH-mut non-codel	IDH mutant with euploid 1p19q
IDH-O	malignant oligodendrocyte-like, IDH-mutated with chromosome 1p19q intact
IDH-wt	IDH wild-type
LGG	low grade gliomas (WHO grade II and III)
M1–M6	methylation subtypes
ME	mesenchymal subtype of grade IV gliomas according to Verhaak et al. (2010)
MGMT	O-6-methylguanine-DNA methyltransferase
MP	methylation pattern
MS	mesenchymal subtype (Sturm et al. (reanalyzed by Hopp et al.))
NGB	neuroglobin
NL	neural subtype of grade IV gliomas according to Verhaak et al. (2010)
NPC	neural progenitor cells
OS HR	overall survival hazard ratio
Ox	oxidative phosphorylation
PG	pre-glioblastoma subtype of LGG (Gorovets et al., 2012)
PN	proneural subtype of grade IV gliomas according to Verhaak et al. (2010)
PRC2	polycomb repressive complex 2
RTK	receptor tyrosine kinase
RTKI	GBM methylation class (Sturm et al.)
RTKII	GBM methylation class (Sturm et al.)
SOM	self-organizing map
TEL	telomerase
TERT	telomerase reverse transcriptase
TF	transcription factors
TME	tumor microenvironment
TMM	telomere maintenance mechanism
