# Supplementary material for: High-Resolution Cartography of the Transcriptome and Methylome Landscapes of Diffuse Gliomas
Source: Cancers (Basel). 2021 Jun 26;13(13):3198. doi: 10.3390/cancers13133198 (PMC8268631; doi:10.3390/cancers13133198)
Supplement: Supplementary file 1 [file cancers-13-03198-s001.zip › LGG_v7_suppl.pdf]

Supplementary Material

High-resolution cartography of the transcriptome and methylome landscapes of diffuse gliomas

Edith Willscher[1], Lydia Hopp[1], Markus Kreuz[2], Maria Schmidt[1], Siras Hakobyan[3], Arsen Arakelyan[3], Bettina Hentschel[2], David T. W. Jones[4], Stefan M. Pfister[4], Markus Loeffler[2], Henry Loeffler-Wirth[1*], Hans. Binder[1*]

[1] IZBI, Interdisciplinary Centre for Bioinformatics, Universität Leipzig, Härtelstr. 16 – 18, 04107 Leipzig, Ger-many; e-mail: edith.willscher@uk-halle.de (EW), lydia.hopp@gmx.net (LH), schmidt@izbi.uni-leipzig.de (MS); wirth@rz.uni-leipzig.de (HLW), binder@izbi.uni-leipzig.de (HB)

[2] IMISE, Institute for Medical Informatics, Statistics and Epidemiology, Universität of Leipzig, Härtelstr. 16- 18, 04107 Leipzig, Germany; e-mail: markus.kreuz@izi-extern.fraunhofer.de (MK), betina.hentschel@iimise.uni-leipzig.de (BH), markus.loeffler@imise.uni-leipzig.de (ML)

[3] Research Group of Bioinformatics, Institute of Molecular Biology of the National Academy of Sciences of the Republic of Armenia, 7 Hasratyan str., 0014, Yerevan, Armenia, e mail: sirashakobyan@gmail.com (SH), aarakelyan@sci.am (AA)

[4] Hopp Children's Cancer Center Heidelberg (KiTZ), Im Neuenheimer Feld 430, 69120 Heidelberg, Germany, e-mail: david.jones@dkfz.de (DTWJ), s.pfister@dkfz.de (SMP)

* Correspondence: binder@izbi.uni-leipzig.de (HB), wirth@izbi.uni-leipzig.de (HLW)

Content

Supplementary Figures 2

Supplementary Tables 24

References 28

Supplementary Figures

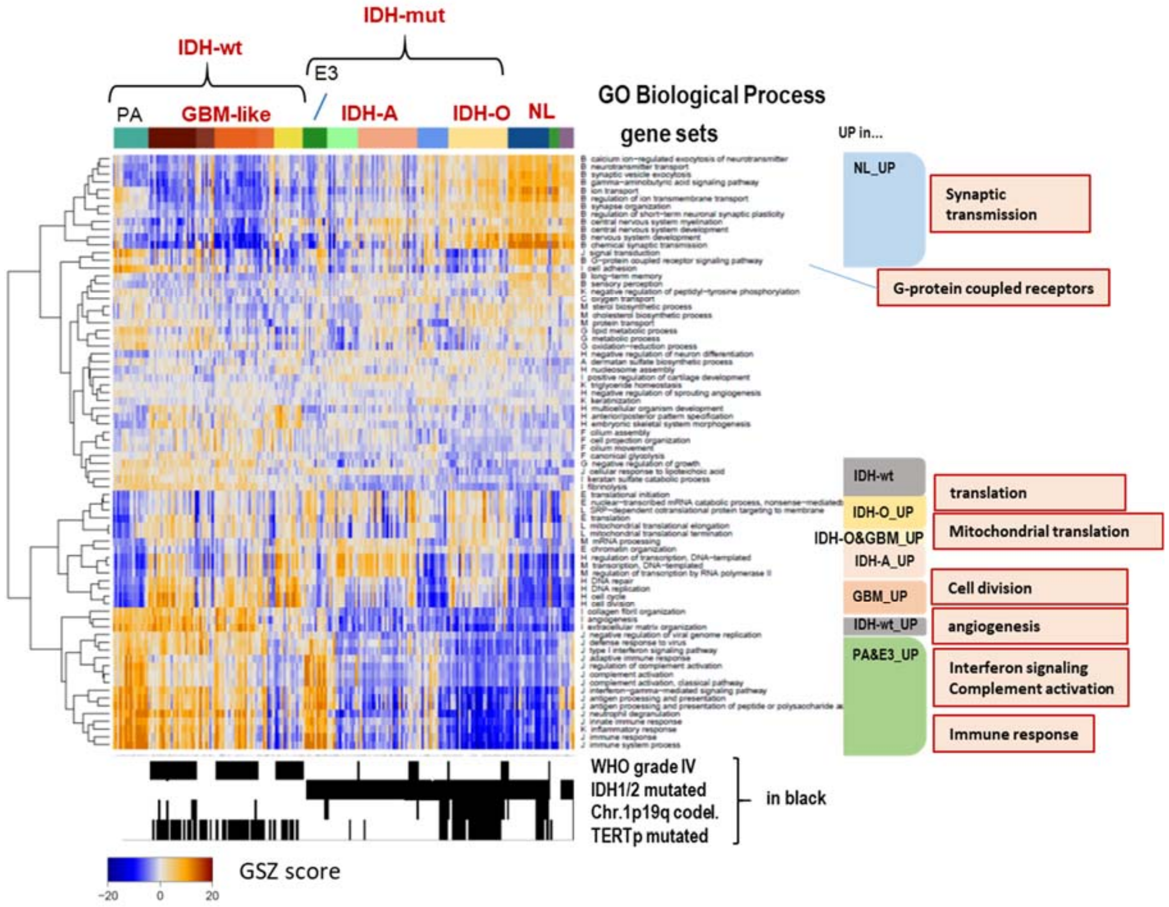

Figure S 1: Functional analysis using the functional category 'Gene Ontology Biological Process: (GO BP) taken from [1]. The heatmap virtually divides into different clusters of genes sets upregulated specifically across the glioma groups (see color bar above the map and genetic characteristics below the heatmap). Clusters are assigned in the right part.

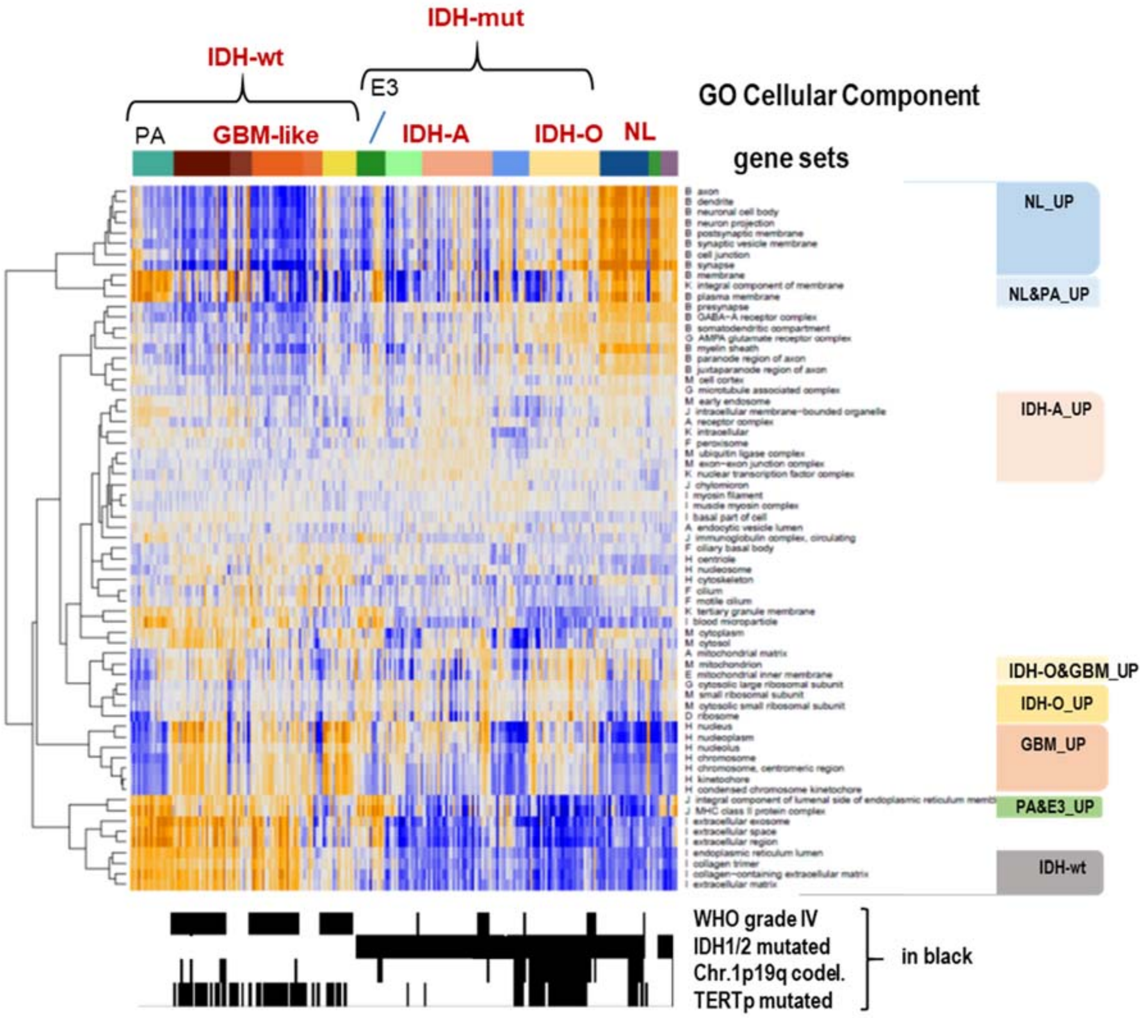

Figure S 2: Functional analysis using the functional category 'Gene Ontology Cellular Component: (GO CC) taken from [1] complements the functional category BP used in Figure S 1.

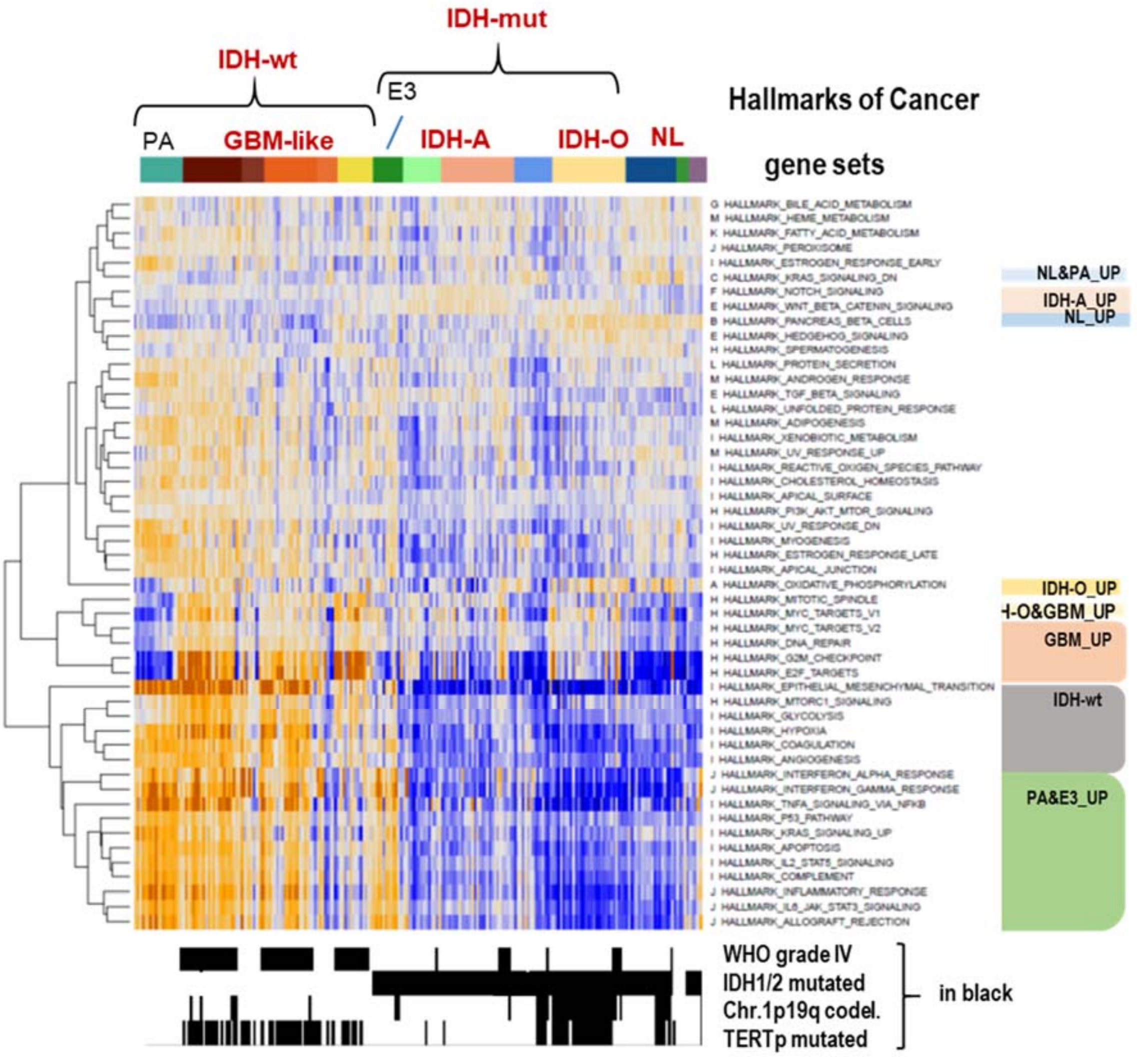

Figure S 3: Functional analysis using gene sets of the functional category ‘hallmarks of cancer’ taken from [2].

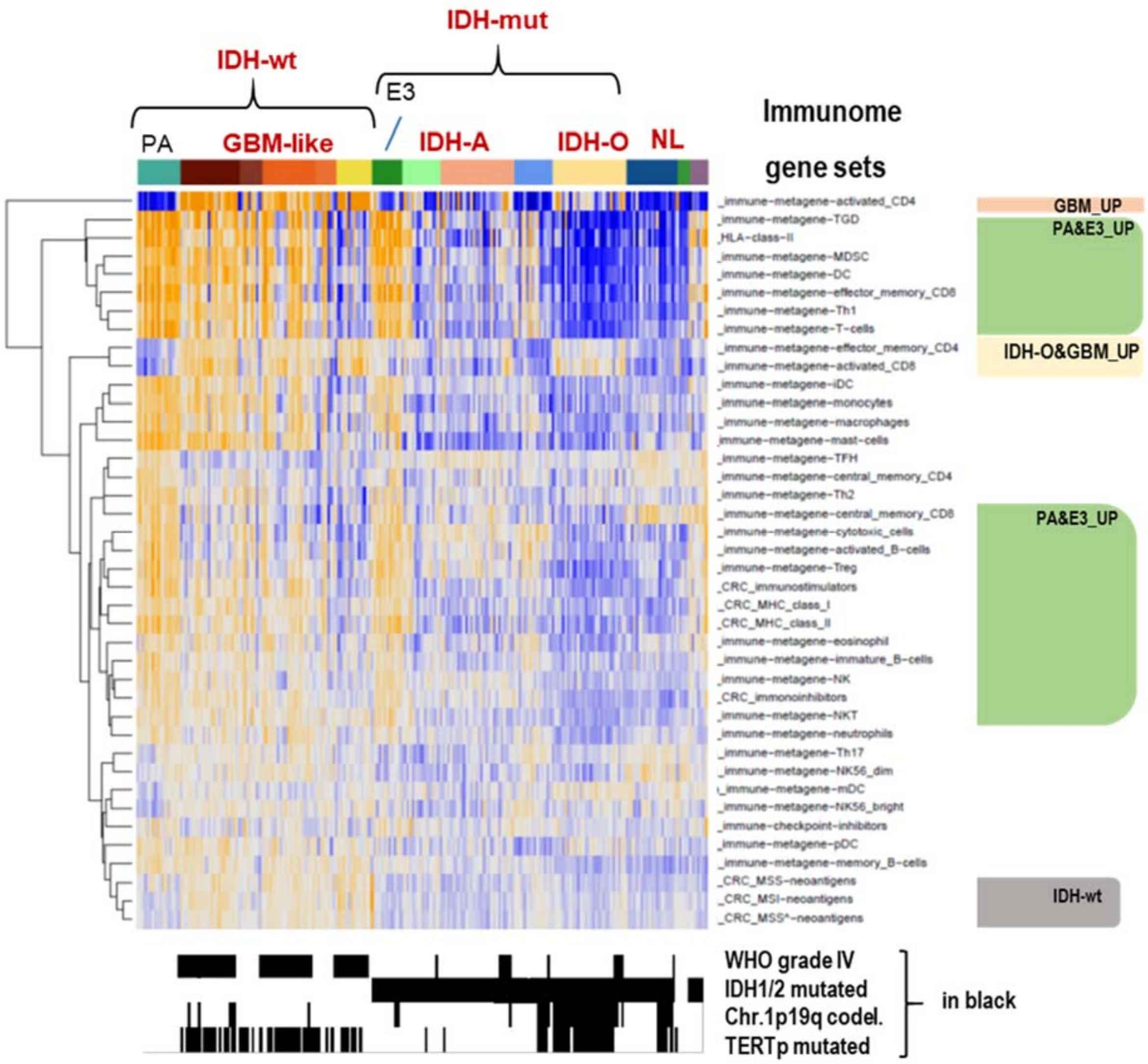

Figure S 4: Functional analysis using gene sets of the functional category 'immunome' taken from [3]. GBM-like IDH-wt gliomas specifically upregulate signatures of neoantigenes taken from colorectal cancers (CRC) together with CD4+ (and, to a less degree CD8+) signatures while other characteristics of immune response (MHC class I and II, HLA class II, MDSC, effector memory CD8 cells, interferon I and gamma signaling, adaptive immunity see PA&E3_UP marks) activate strongly in E3 and PA gliomas. Specific upregulation of immune checkpoint inhibitors is found also in PA and E3. Note that low activity of virtually all immune response signatures is found in IDH-O gliomas.

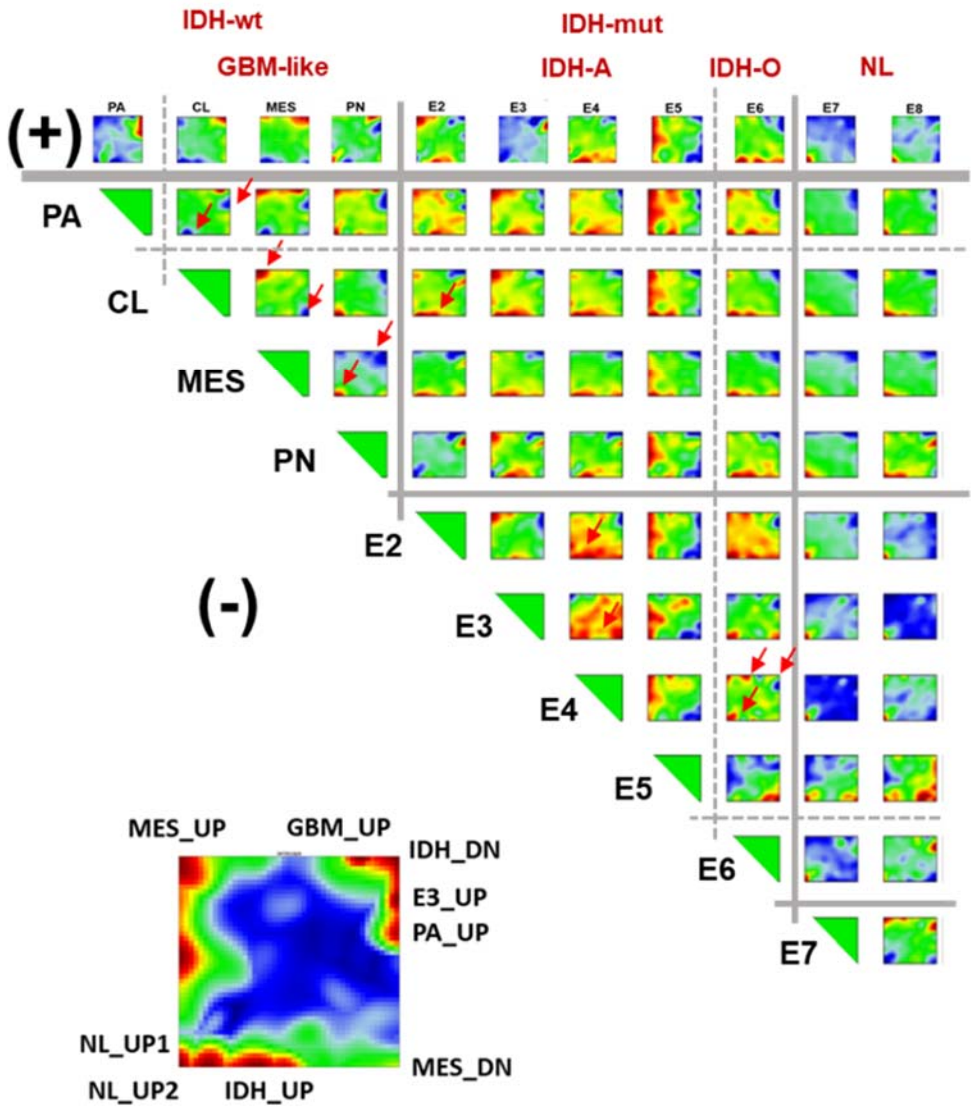

Figure S 5: Pairwise differences between the SOM-expression portraits of glioma indicate group-specific transcriptome patterns. The summary map in the part left below provides an overview about the major expression modes assigned in the main paper**Fehler! Verweisquelle konnte nicht gefunden werden.**. Arrows indicate spot areas referring to the respective differences. For example, difference portraits with respect to PA (second row from above) shows the PA_UP spot as a specifics of PA tumors.

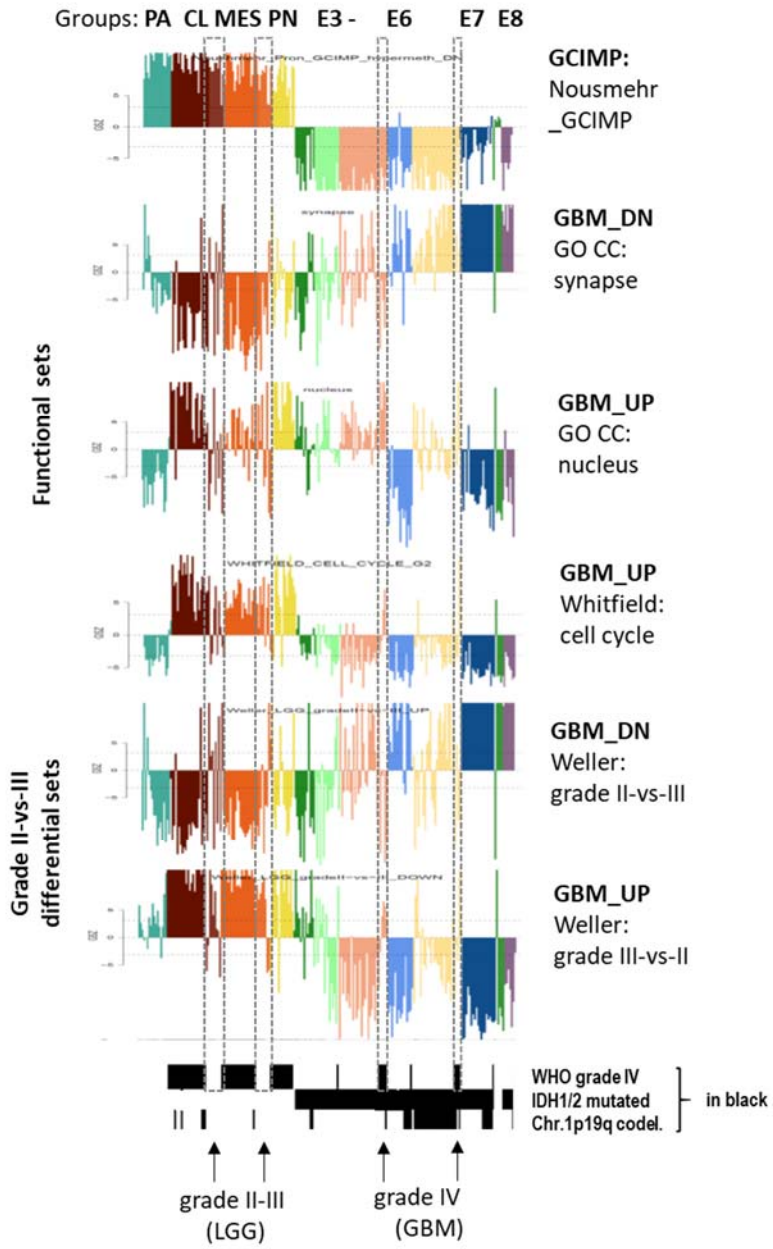

Figure S 6: Comparison of selected expression gene set profiles between grade IV (GBM) with grade II-III (LGG) gliomas. Gliomas were sorted subtype-wisely, i.e. from the left to the right IDH-wt first, then IDH-mut followed by NL of mixed composition. In each group WHO-grade II and III (LGG) and grade IV were grouped together as shown by the black-white bar below the profiles. The dashed vertical lines include IDH-wt LGG in the left part and IDH-mut GBM in the right part, i.e. the minority fractions in each of the respective groups. The GCIMP-profile clearly splits the cases into IDH-wt (upregulated) and IDH-mut (downregulated) virtually independent of their WHO-grade. GBM_UP ('nucleus', Cell cycle' [4]) and GBM_DN ('synapse') profiles refer to gene sets showing over- or under-expression of grade IV (GBM) compared with LGG in each of the groups, respectively. Similar relations were observed for gene sets of differential expression between WHO-grade II and III gliomas [5]. These results indicate a continuous loss of healthy brain function and the gain of cancer-related features with increasing grade of the tumors from WHO II to IV. These results also support the view that IDH-mut gliomas (grade II-IV) and IDH-wt gliomas were recognized as different clinical and genetic entities where IDH-wt have usually much more aggressive clinical behavior [6].

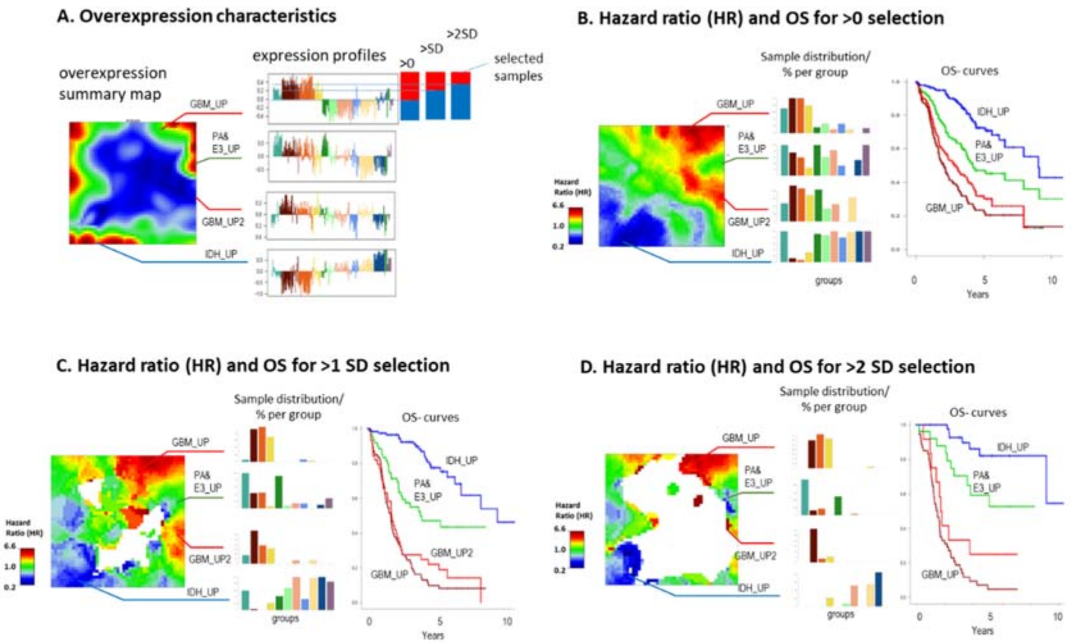

Figure S 7: Prognostic maps: A) The overexpression summary map visualizes gene clusters (spots) showing high expression levels in any of the subtypes as illustrated by selected expression profiles referring to different areas of the map as indicated. Glioma-patients were selected using different expression thresholds (>0, >SD, >2SD, SD…standard deviation) in each of the metagenes. Because of centralization of expression values these thresholds refer to mean expression of each gene averaged over all samples. Each metagene-pixel of the prognostic maps shown in part B- D was then color-coded according to the hazard ratio (HR) of the selected gliomas from red (high HR) to blue (low HR). B)- D) Prognostic maps obtained for different thresholds for sample selection. With increasing threshold, the number of selected samples in each of the metagene-pixels decreases giving rise to more specific subgroup selection (see the barplot showing the sample frequency distribution across the subgroups) and wider spread of the overall survival (OS) curves better indicating prognostic differences of the genes taken from the different areas. On the other hand, a too restrictive selection criterion reduces the number of samples selected and the associated significance of the HR data. White regions in the HR-maps refer to situations where no sample meets the > 1SD or > 2SD criterion, respectively. Independent of threshold selection the HR maps all reveal similar properties, namely worse prognosis if GBM-characteristic genes upregulate and good prognosis if IDH-mut typical genes upregulate.

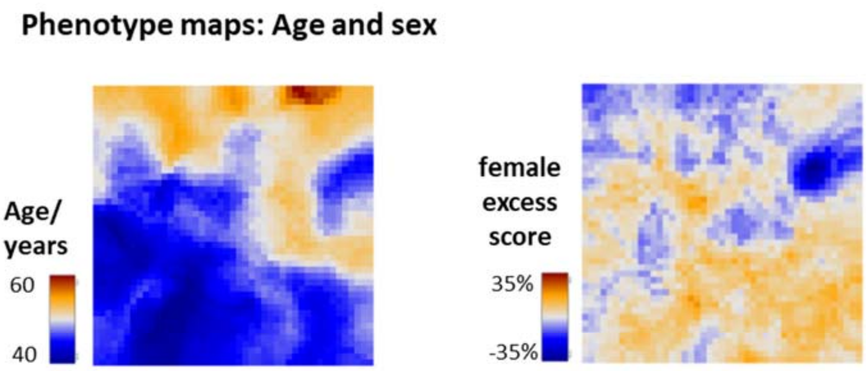

	PA	CL	MS	PN	E3	E2	E4	E5	E6	E7	E8
Age years	25± 15	58± 8	61± 9	53± 17	42± 11	46± 15	34± 10	44± 12	48± 13	37± 9	51± 11
% fem	41	44	31	47	42	40	38	33	45	25	0

Figure S 8: Phenotype maps visualize associations between the SOM-transcriptome landscape and age at first diagnosis (left part) and sex of the patients (right part). Associations were calculated as mean age and percentage of women among glioma patients in each of the metagene-pixels whose expression exceeds the chosen threshold (0, see Figure S 8). Comparison of the age-map with the HR-map in Figure S 8 reveals that bad prognosis associates mainly with higher age of the patients, which, in turn, is observed for IDH-wt gliomas compared with IDH-mut (and especially IDH-A compared with IDH-O) and in contrast to relative young PA-patients. Female patients enrich in gene expression patterns upregulated in IDH-A and CL but not PA and MES gliomas. The table lists the mean age and percent of women in each of the groups.

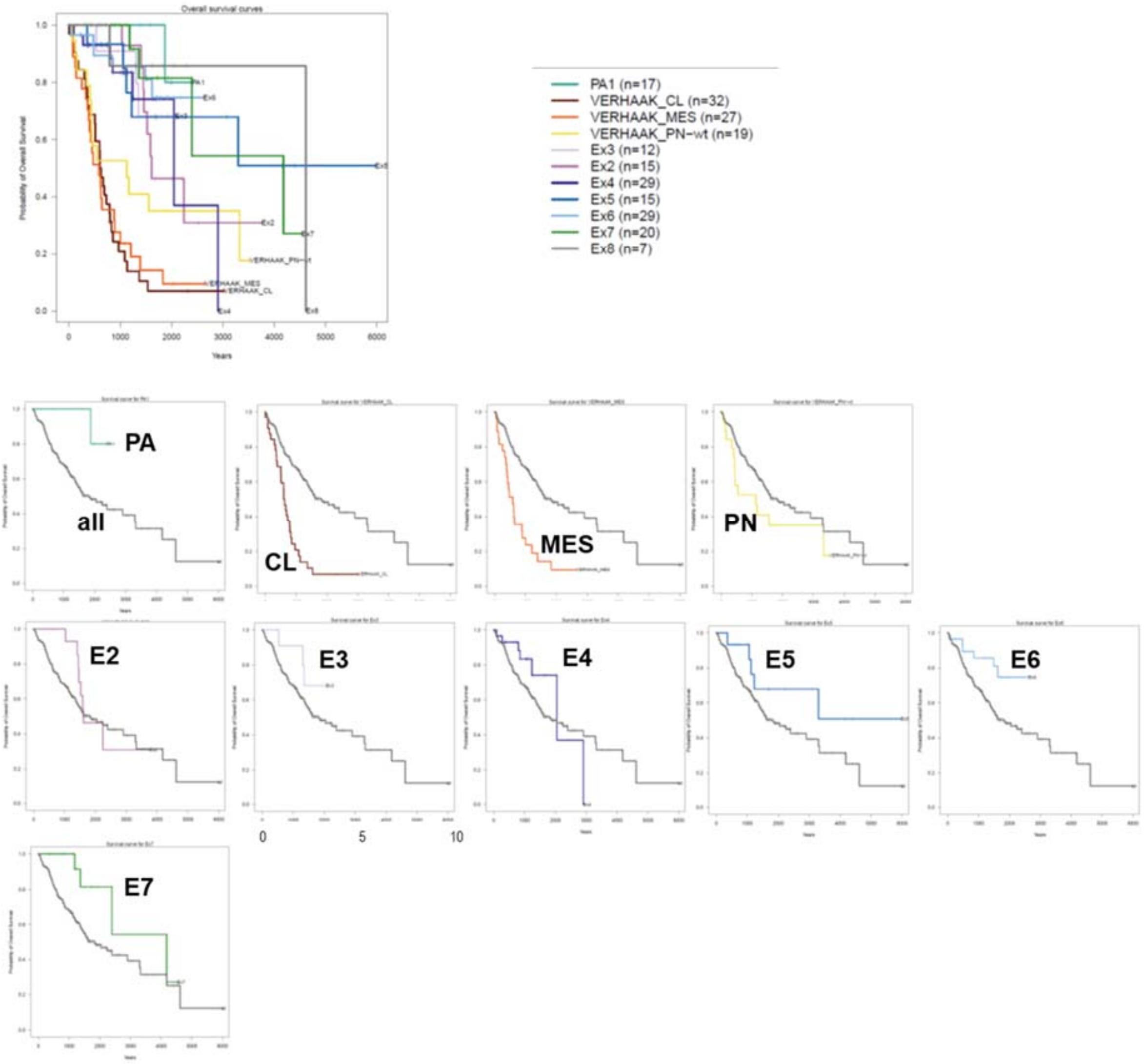

Figure S 9: Overall survival (OS) curves of the glioma groups studied. A) OS of all groups in one plot for comparison and, B) separate plots for each group compared with the mean OS curve for all tumors studied. The GBM classes CL and MES associate with poor prognosis compared with PN-GBM and IDH-A LGG show better outcomes. PA and IDH-O reveal best prognosis. Details are provided previously [5, 7, 8].

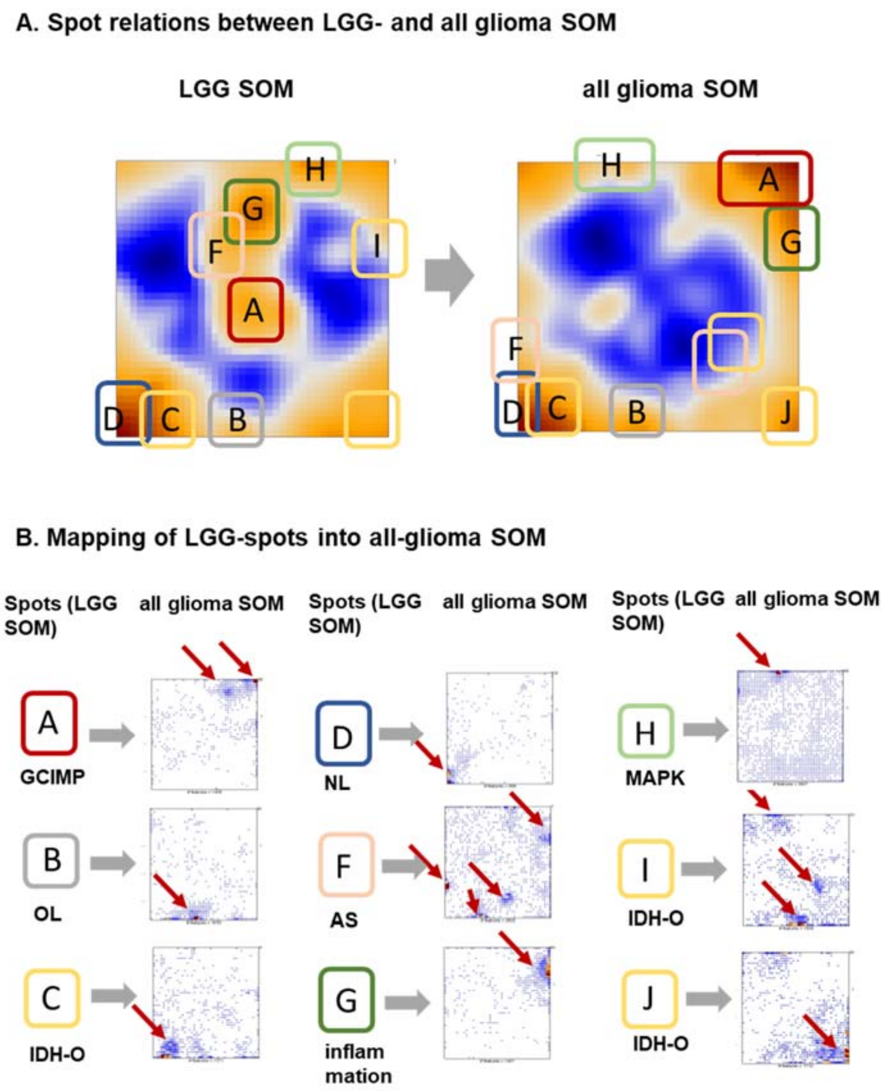

Figure S 10: Mutual mapping of the spot-clusters between the ‘all-gliomas’ and ‘LGG-only’ SOMs. A) The variance maps of both SOM indicate areas of highly variant expression in maroon color and areas of invariant expression in blue. Spots are labeled by letters as used in the LGG-SOM. Spot position and arrangement differ between both SOM, can be assigned however in an one-to-one relation one to another in almost all cases. B) Spot-genes of the LGG-SOM (assigned by the respective letters and the major spot function) are mapped into the ‘all-glioma’ SOM. Most spots virtually transform in a one-to-one fashion as indicated by the accumulation of the respective spot genes in one area of the ‘all-glioma’ map (see arrows). Exceptions are spots F and I representing marker spots of IDH-A and IDH-O gliomas, respectively. They decompose into three to four spot areas in the ‘all-glioma-map’ meaning that the larger diversity of IDH-wt gliomas in this data resolves more transcriptional states and thus increases specificity/selectivity of selected transcriptional modes. For example, spot F splits into modules due to (healthy) astrocytes (AS), neoplastic astrocytoma cells (IDH-A), immune bystander cells and presumably neoplastic oligodendroglioma (OL) cells.

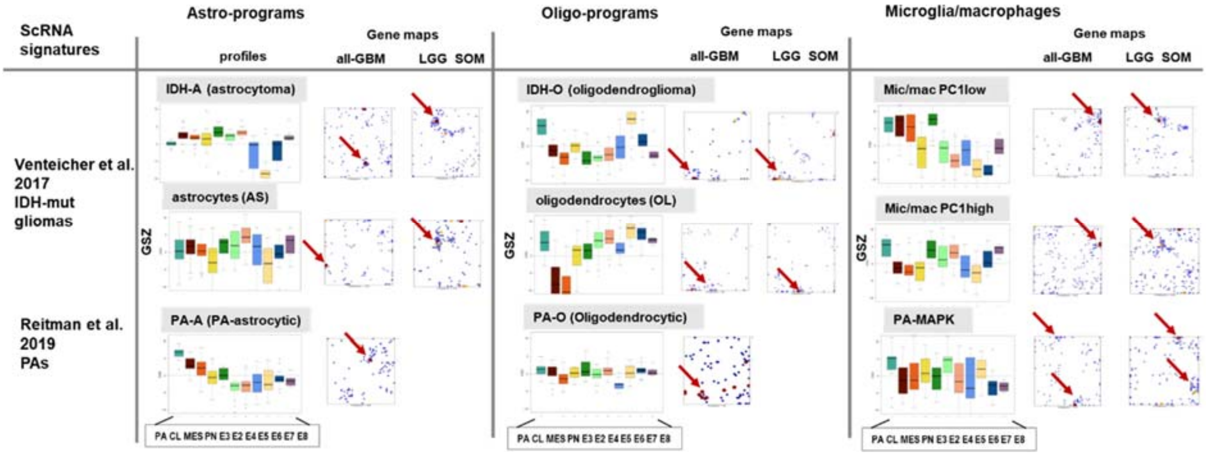

Figure S 11: Mapping of single cell glioma signatures extracted from IDH-mut gliomas [9] and pilocytic astrocytomas (PA) [10] into the 'all-glioma' and LGG SOM. The single cell signatures characterize different cellular components constituting IDH-mut and PA gliomas such as (benign) astrocytes (AS) and oligodendrocytes (OL), cancer cells referring to astrocytomas (IDH-A, PA-A), oligodendrogliomas (IDH-O, PA-O) as well as microglia/macrophages (mic/mac) and cancer cells showing activated MAPK-signaling in PA (PA-MAPK). The profiles indicate group-specific activation and deactivation while the gene maps show accumulation of the genes of the respective sets in distinct areas of the all-GBM and LGG SOM (arrows). These maps enabled us to assign the spots in the bulk-tumor samples used in this work (Figure S 10). For example, spot F in the LGG-SOM splits into four spot-areas in the all-glioma SOM. The single cell signatures assign them to AS, astrocytoma cells (IDH-A) and also to mic/mac cells (specific to IDH-A) and OL –resembling cells. Spot I (IDH-O) combines OL, IDH-O and PA-MAPK characteristics. Interestingly PA-MAPK single single cell signatures resembles the activation patterns of E2 gliomas while the microglia/macrophage signatures accumulates in spots upregulated in E3. Astrocytic (healthy), IDH-mut astrocytoma and PA-astrocytoma signatures are similar, but not identical.

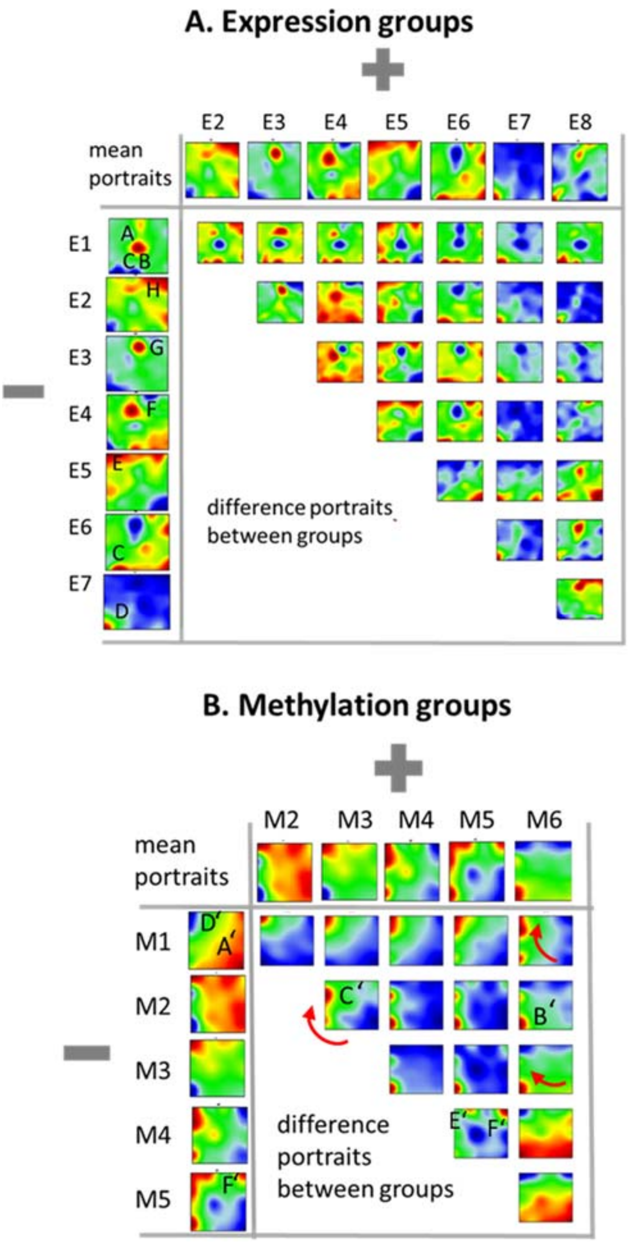

Figure S 12: Pairwise difference SOM portraits between the mean portraits of the expression (part A) and methylation (part B) subtypes reveal subtype specific and shared spot patterns between the subtypes. A) Expression spot A (GCIMP-genes) is specifically upregulated in E1 while spots B and C are downregulated. E3 shows strongest expression of the inflammatory spot G among all subtypes, spot F (astrocytes) is characteristic for E4 and spot D (healthy brain) for E6. B) Methylation spot A’ is specifically hypermethylated in M1 while spot D’ (GCIMP) switches antagonistically showing increasing hypermethylation in M2 – M5. Spots C’ (‘keratinization’) and B’ (*GPCR*) are commonly red in almost all difference portraits meaning that methylation progressively decreases from M6 to M1 where strongest effect on C’ is observed M3. M5 shows specific hypermethylation of E’ and F’.

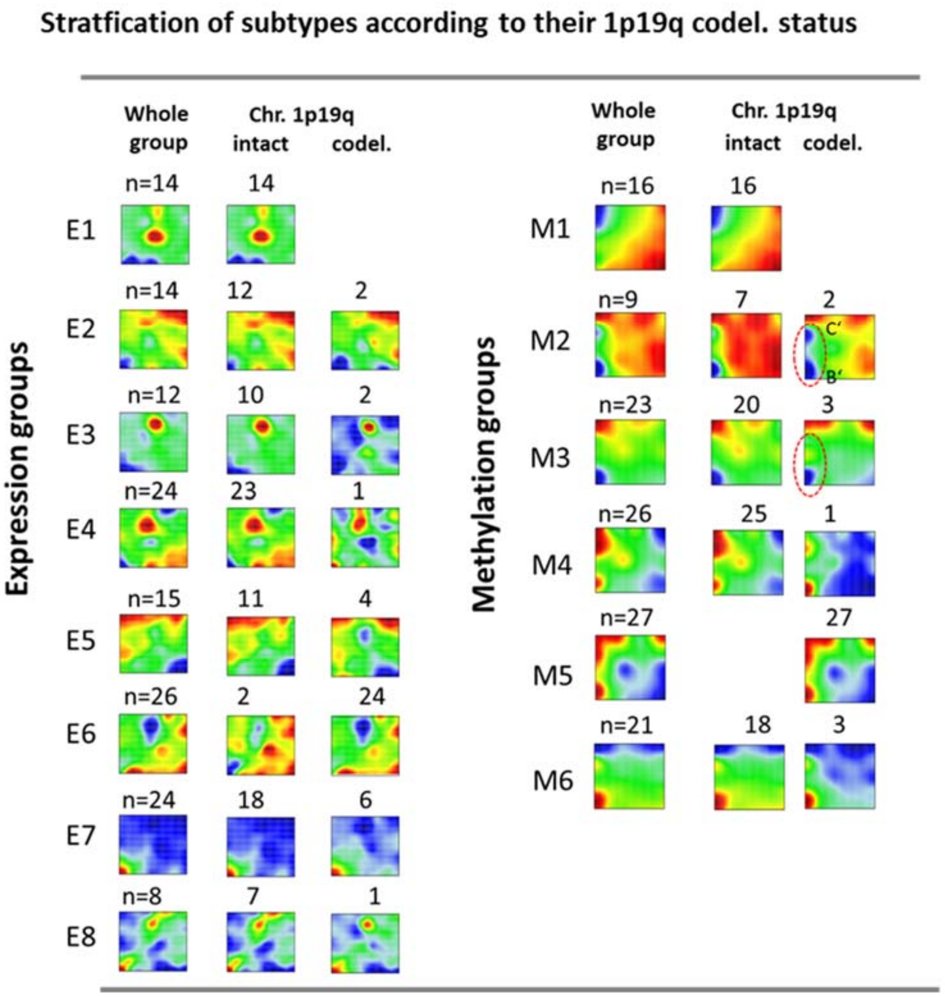

Figure S 13**:** Stratification of the E- and M-subtype portraits into mean portraits of Chr.1p19q-codeleted and -intact tumors: Chr. 1p19q co-deletion strongly accumulates in groups E6 and M5, which this way reflect the 'canonical' expression and methylation landscapes of the majority of Chr. 1p19q codeleted tumors. On the other hand, about 5 – 20% of the gliomas of the other groups also carry the Chr. 1p19q co-deletion. In these tumors the Chr. 1p19q co-deletion has only small effect on the molecular landscapes, which are obviously dominated by the other factors discussed in the main paper. Genes in spots B' and C' hypermethylate in M5 collecting solely Chr. 1p19q co-deleted tumors (right part of the figure). These spots show hypomethylation independent of their Chr. 1p19q codeletion status in other groups (red dashed ellipses). Hence, Chr. 1p19q codeletion associates predominantly with hypermethylation of spot B' but also with hypomethylation in a minority fraction of tumors (see also Figure S 14).

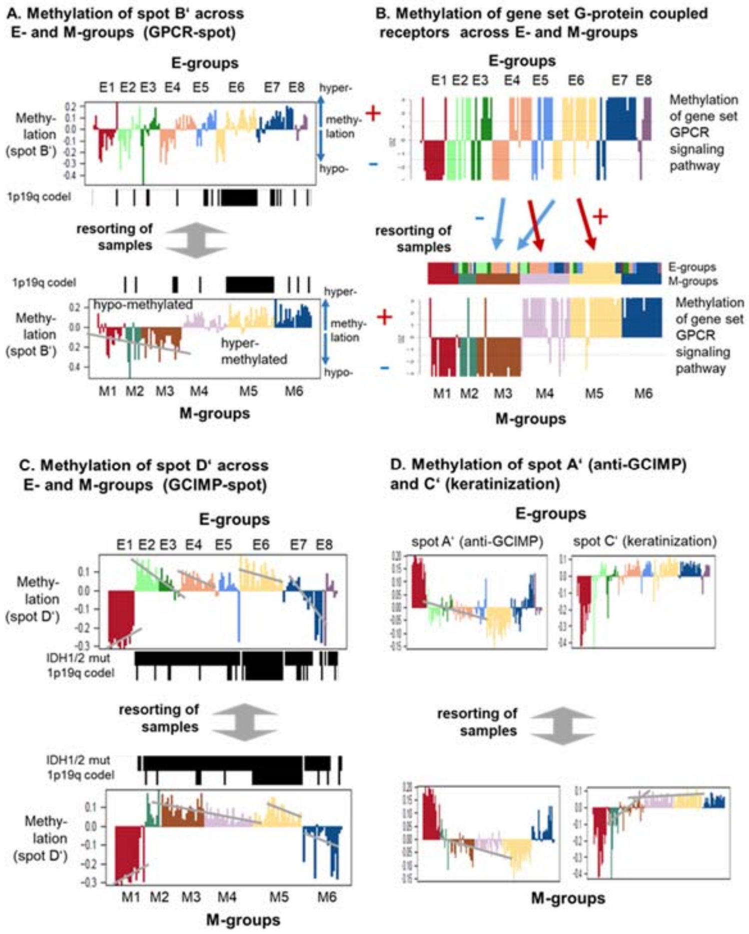

Figure S 14: Resorting of tumors between E- and M-groups owing to different methylation of the olfactory subgenome reflects partial decoupling between transcriptional programs and the global DNA-methylation patterns: A) Gliomas divide into M-groups showing strictly hyper (M4 – M6) or hypo (M1-M3) methylation of spot B' (strongly enriched with genes of the G-protein coupled receptor/GPCR, olfactory subgenome [8]). Hence, methylation is clearly bimodal in M-groups, namely it splits cases between M1-M3 (hypomethylated with respect to mean methylation) and M4-M6 (hypermethylated). In contrast, E-groups all show mixed composition of hypo-/hypermethylated tumors where the relative amount of hypomethylated (spot B'/olfactory subgenome) tumors progressively increases from E7 to E1. B) The GSZ-methylation score of the gene sets 'GPCR-signaling' (GO biological process) clearly shows this resorting of tumors between M- and E-groups and indicates its direct relation to the methylation of the olfactory subgenome in LGG. The arrows indicate these re-sortings of hypo- (blue) and hyper-methylated (maroon) tumors in E4 and E6 into M3 and M4&5, respectively. Note that majority of Chr. 1p19q co-deleted tumors accumulate in M5 showing hypermethylation of B'-genes while a smaller fraction is hypomethylated and group into M1-M4. C) and D) Methylation of spots D' (GCIMP-profile) and A' (anti-GCIMP) show virtually similar methylation trends in the E- and M-groups mostly corresponding to the IDH-mut status of the tumors. Spot C' (keratinization) shows similar decoupling of methylation and expression as observed for spot B'. Hence, part of methylation spots shows correspondence between M- and E-groups (e.g. GCIMP-spot D' governed by IDH-mut) while others (e.g. GPCR-spot B') do not. The whole transcriptome and methylome landscapes are superpositions of the respective spots giving rise to partly diverging class-assignments if classified independently (see also the E- and M-class color bars in part B). The grey lines serve as guide for the eyes to mark trend of sample's methylation. Note that samples in each subgroup were sorted from high-to-low overall methylation from the left to the right.

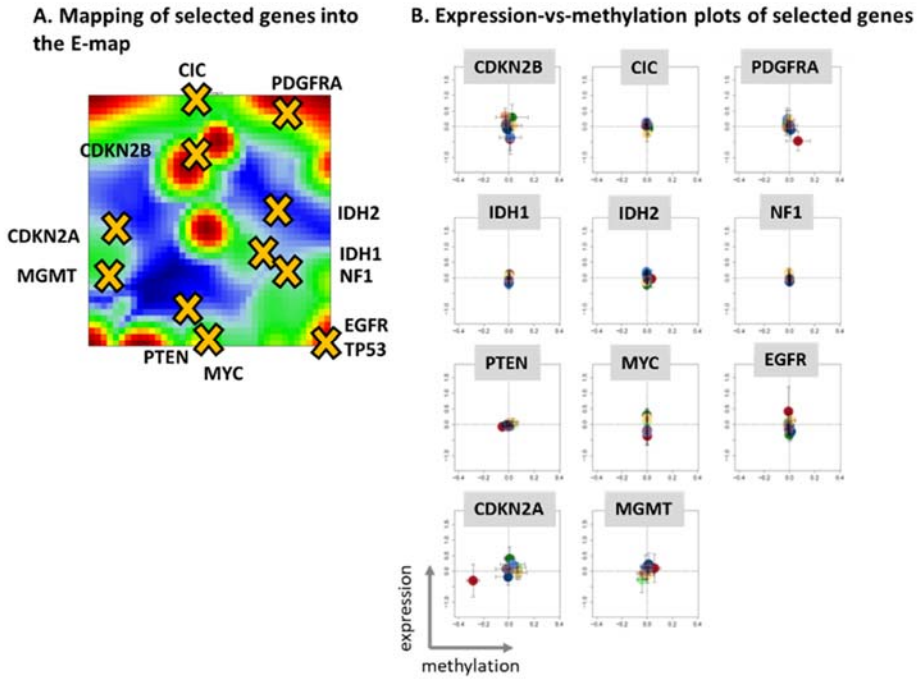

Figure S 15: Selected glioma key genes and the correlation between their expression and DNA-methylation levels in the E-groups. Expression of most of these genes (except CDKN2A and PDGFRA) are only weakly affected by DNA-methylation. This is reflected by the observation that these genes deplete in areas of spots A (GCIMP), D (synapse), F (astrocytes) and G (inflammation).

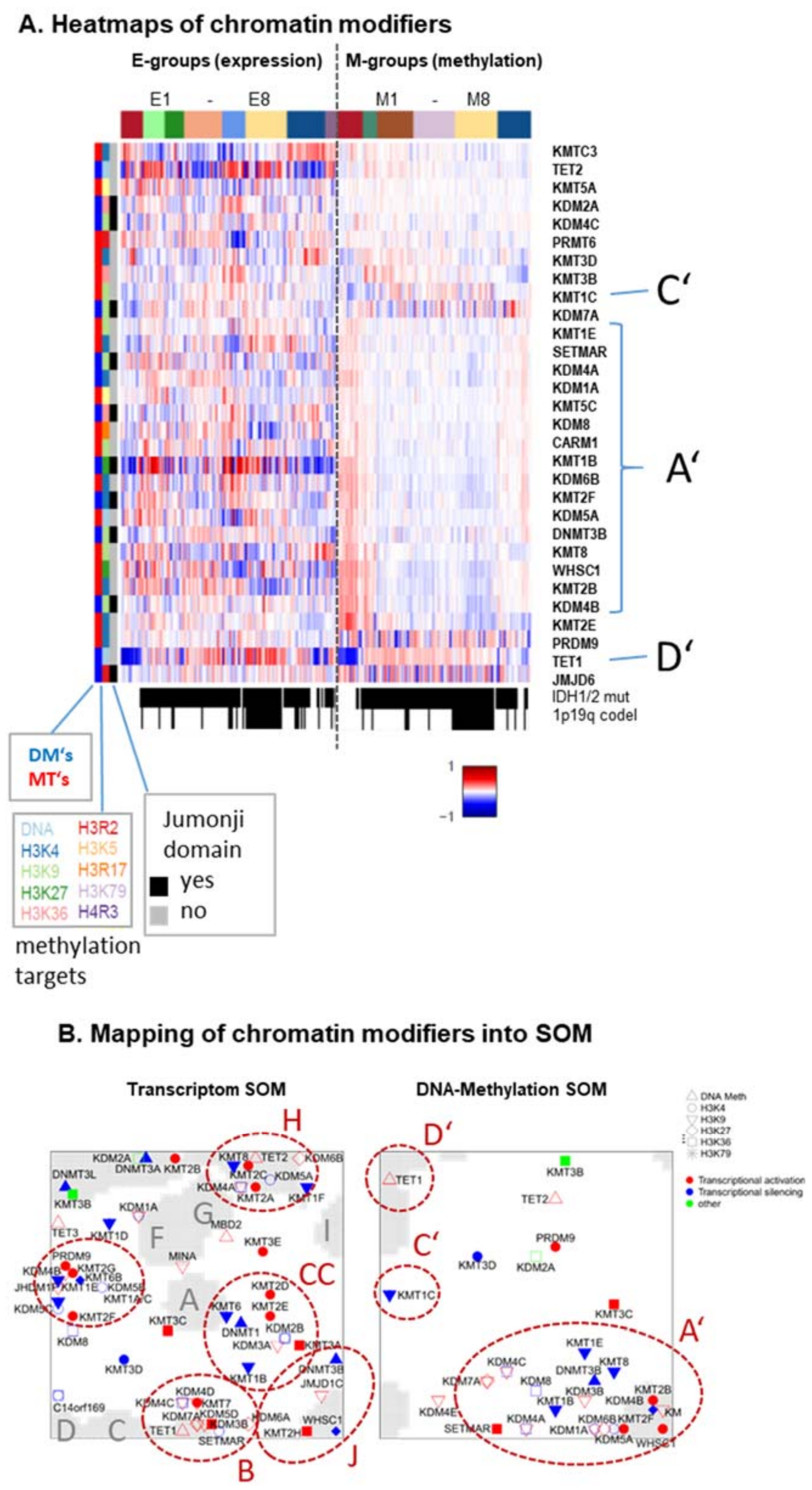

Figure S 16: Gene expression and DNA-promoter methylation of chromatin modifying enzymes including methyl-transferases (MT) and demethylases (DM) of methyl groups linked to of DNA CpG's, lysine (symbol 'K') or to arginine ('R') side chains of histones, mostly at the H3 histone subunit: A) Combined gene expression (left side, log-expression scale) and gene promoter-methylation (right side, log_M scale) heatmap of chromatin modifying enzymes in the LGG expression/methylation groups. The type of enzyme (MT or DM), the methylation targets (DNA, histone lysine or arginine side chains at H3) and the Jumonji-domain type (activity of Jumonji-domain containing enzymes is repressed by the onco-

metabolite 2-HG produced in IDH-mut but not in IDH-wt gliomas). B) Mapping of expression and methylation of the enzymes into the expression and methyation SOM. The dashed circles indicate the spot areas accumulating enzyme-coding genes. Spots with depleted presence of enzyme genes were assigned with grey letters (spots A, F, G, C, D, I).

Overall, we took into account the gene expression levels of more than thirty chromatin modifying enzymes in the LGG data. The methylation profiles show predominantly anti-GCIMP (A' spot-cluster) characteristics, which is characterized by hypo-methylation in IDH-mut and hypermethylation in IDH-wt gliomas. The expression SOM reveals depletion of genes in spots A and D, which is compatible with the anti-GCIMP methylation profile because it corresponds to transcriptional repression of genes in the IDH-wt groups E1 and E7. The enzyme-genes heterogeneously distribute among the other spots of the expression map reflecting a subtle activation/deactivation patterns in the different IDH-mut groups. Depletion of genes is also observed in spots C (upregulated in E7/NL and E6), F (up in E4, astrocytes), G (up in E3, inflammation) and I (up in E6), which indicates the lack of association of transcriptional activation of genes coding chromatin-modifying enzymes with those processes/groups. On the other hand, accumulation of chromatin modifiers is observed in spots B (up in E4 and E6), CC (proliferation and cell cycle activity), H and J (up in E2 and E6), which reflects their upregulation in the respective subtypes and processes. For example, we find DNMT1 and KMT6 (alias EZH2) in the proliferative spot CC associating with high cell cycle activity. DNMT1 codes a methyltransferase maintaining DNA methylation after cell divisions and contributing to double strand DNA break repair [11, 12, 13]. KMT6 is part of PRC2-complex. It methylates histone lysine side chains at H3K9 and H3K27. Changes of its expression promote malignant transformation, e.g. by repressing anti-proliferative and differentiation-inducing programs, proliferation checkpoint genes and by modifying cell-fate decisions upon cell differentiation [14, 15, 16, 17]. The upregulation of DNMT1 and KMT6 in parallel with cell cycle activity can be rationalized by the need for activated maintenance for restoring DNA and histone methylation marks in highly proliferative cells after each cell division. The de-novo methyl-transferase DNMT3B has impact for tissue-development and cell-differentiation. It locates in spot J, which upregulates especially in IDH-O. DNA demethylases of the TET-family play an important role in regulation of DNA methylation. Their post-transcriptional inactivation, e.g. via inhibition by the oncometabolite 2-HG in IDH-mut gliomas, essentially contributes to the establishment of hypermethylation phenotypes such as GCIMP [18]. TET1, 2 and 3 locate in/near spots B, H and E, respectively, showing diverse up- and downregulation in IDH-A (E2 and E4) and IDH-O (E6) gliomas. The TET1-gene promoters show the GCIMP-methylation profile (spot D') which suggests a 'repression of DNA-demethylation via promoter methylation of TET1'-feedback mechanism and thus self-amplification of GCIMP-methylation. TET2 shows differential expression and methylation especially between E4 (IDH-A) and E6 (IDH-O), which suggests a role in the differential epigenetic regulation of cellular programs between astrocytomas and oligodendrogliomas. Spot B accumulates predominantly enzymes associating with transcriptional activation (colored in red) affecting especially E2 and E4 (IDH-A) and E6 (IDH-O). Among them are KMT7 (alias SETD7; it methylates H3K4 and also the non-histone protein p53/TP53 at K372) and KDM7A (a H3K9 and H3K27 demethylase with role in brain development) [17]. The H3K4 demethylase KDM1A (alias LSD1) is found near the otherwise depleted spot F (up in astrocytoma). It de-methylates H3K4me3 in gene bodies of inactive genes this way maintaining genes in their bivalent-active or repressive state. Overall, we a find a predominant anti-GCIMP methylation profile of the epi-enzymes studied which however transforms into a heterogeneous transcriptional pattern, where expression of epi-enzymes either associate with increased cell cycle activity of cancer cells or they seem to play roles in differences in epigenetic regulation between IDH-O and IDH-A gliomas.

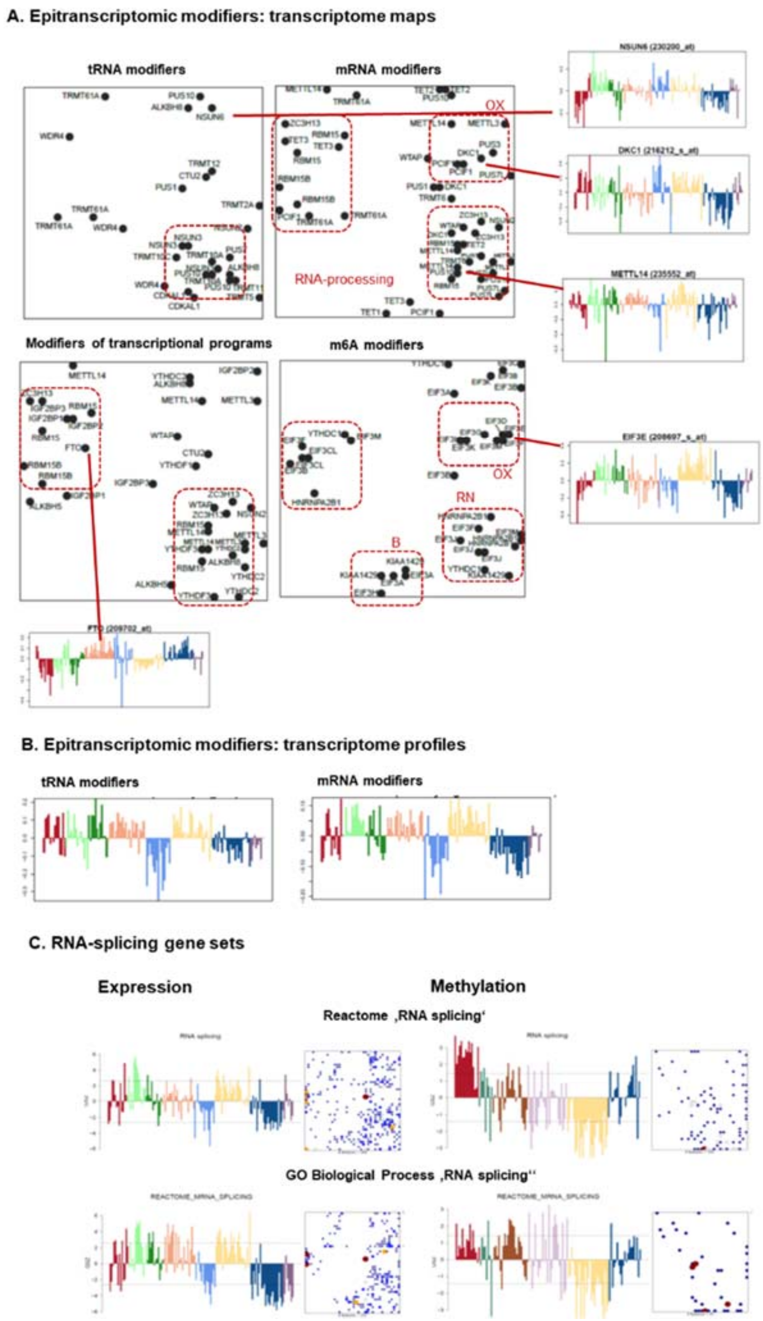

Figure S 17: Expression of genes coding epi-transcriptome modifying enzymes: A) Expression maps of genes coding epi-transcriptome modifying enzymes. Genes were taken from the reviews [19, 20] and assign writers, readers and erasers of post-transcriptional RNA modifications (e.g., methylation and isomerization of RNA bases A, U and G, mostly N6-methyladenosine, m6A). Modifications affect tRNA this way modulating translation (upper left figure, see Fig. 1a in [19]) and mRNA which modulates transcriptional programs (upper right, see Fig. 1b in [19]). RNA-modifiers affecting transcriptional programs taken from [19] (see Fig. 2 there) and writers, readers and erasers of m6A (see overview given in [20]) are shown in the left and right lower maps, respectively. Gene expression profiles are shown for FTO, METL14, EIF3E, NSUN6 and DKC1 located in different areas of the map which accumulate RNA modifiers. They associate with functions such as oxphos (OX), RNA-processing (RN) and spot B which upregulates in IDH-O gliomas. Regions accumulating GCIMP genes (spot A), immune- (G) and healthy brain (D) functions are depleted from RNA-modifiers. This effect was also observed for chromatin modifiers (Figure S 16). B) The integral expression of all genes considered as tRNA or mRNA modifiers taken from part A revealed concerted upregulation in all glioma subtypes (except E5) compared with E7, with slightly stronger effects in the IDH-mut subtypes (E2, E3, E4, E6) compared with IDH-wt (E1). C) Expression (left part) profiles and gene maps of the gene sets ‘RNA splicing’

(Reactome and GO) resemble profiles and maps of the epi-transcriptome genes shown in part A and B. This similarity suggests that epi-transcriptome modifiers are embedded into the RNA-processing machinery of the cells becoming jointly activated in gliomas. The respective DNA-methylation profiles (right part) show anti-GCIMP-O characteristics suggesting partly repressive effect of DNA-methylation of gene promoters on gene expression.

Previous studies reported that the m6A demethylase ALKBH5 is highly expressed in glioblastoma and sustains the proliferation of patient-derived glioblastoma cells. Also FTO another m6A demethylase is highly expressed in patients with acute myeloid leukaemia promoting cell proliferation and survival, acting partly in anti- concert with MTTL3 or METTL14 acting as m6A methyltransferases (see [19, 20] and references cited therein). Notably, like TET and Jumonij domain containing enzymes, FTO is inhibited by the oncometabolite 2HG produced in IDH-mut gliomas leading to increased m6A-levels in IDH-mut tumors which reflects direct coupling between glioma driver mutations and epi-transcriptomics. Interestingly, modifiers of the 'OX'-cluster differentially regulate between E6 (UP) and E1 and E4 (down) while FTO (and other modifiers from this cluster) show antagonistic regulation (E6 down; E1 and E4 up), which implies prognostic impact due to different prognosis of E6 (good prognosis) and E1&E4 (worse prognosis) in correspondence with a recent publication reporting prognostic epi-transcriptome markers [21].

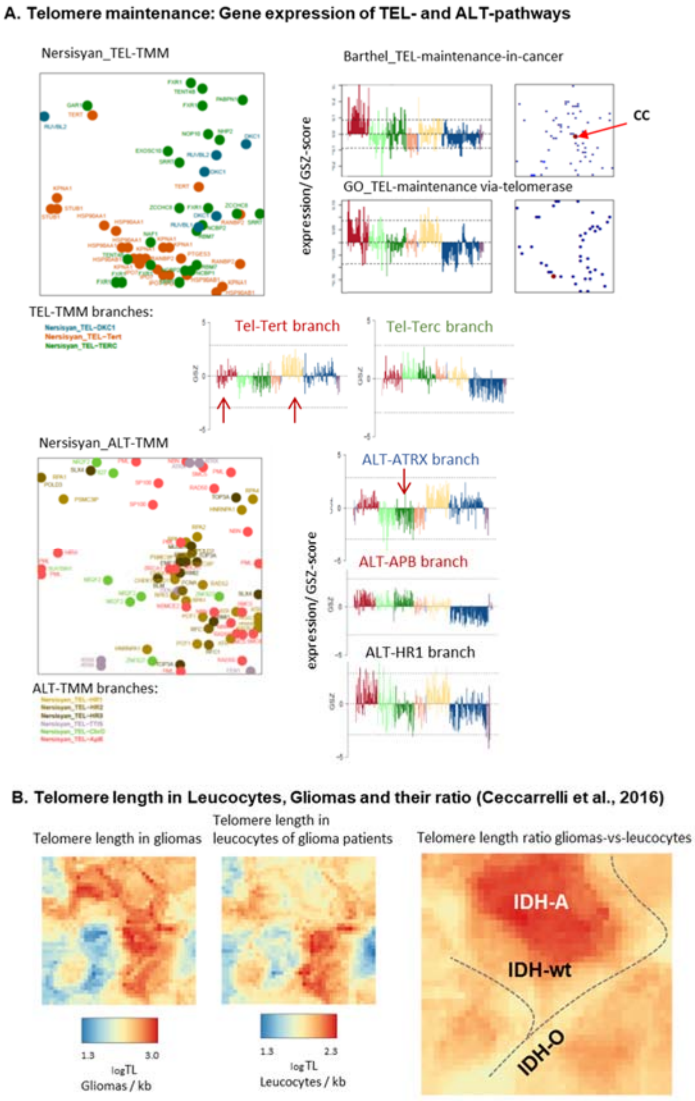

Figure S 18: Telomere maintenance in gliomas: A) Map of genes involved in telomere maintenance mechanisms via telomerase activation pathway (TEL-TMM) taken from [22], [23] and from gene ontology (GO). The TEL-TMM splits into three branches (see [22] for details), where Tel-Terc and Tel-DKC1 genes (green and blue dots) arrange along a vertical 'branch' in the map which associates with upregulation in tumors compared with specimen of the NL type (E7), especially in IDH-A (see the Tel-Terc profile). In contrast, the Tel-Tert genes (red dots) arrange along a more horizontal 'branch' and associate with selective activation in IDH-wt/E1 and IDH-O/E6 gliomas (see arrows in the Tel-Tert profile). This effect corresponds to Tert promoter mutations which enrich in IDH-wt and IDH-O gliomas and strongly activate transcription of the (otherwise inactive) Tert-gene [23, 24]. Also the genes of the gene set 'TEL_maintenance-via-telomerase' show these two branches in the gene map and selective activation of IDH-wt and IDH-O gliomas. The gene set 'Bartels_TEL-maintenance-in-cancer' was extracted from a series of cancer entities with activated telomerase [23]. It enriches genes whose expression correlates with CC (cell cycle function) and immune functionalities (spot G), which leads to higher expression in E1 and E3. In contrast to IDH-O gliomas IDH-A typically show no mutations of the Tert genes but instead carry deactivating mutations of ATRX which promote alternative lengthening of telomeres (ALT-) TMM. Indeed, gene expression of the ATRX-branch of the ALT-TMM is strongly downregulated in IDH-A gliomas (see arrow). Other branches of the ALT-TMM [22] either upregulate in concert with inflammatory processes in E1, E2 and E3 (ALT-APB: ALT-associated promyelocytic leukemia nuclear bodies) and cell cycle activity in E1 and E3 (ALT-HR: homologous recombination). As the major effect, transcriptomic data thus support mutually exclusive activation of TERT-TMM in IDH-wt and IDH-O and of ALT-TMM in IDH-A. B) Phenotype-association maps between telomere lengths in gliomas, the glioma patients blood (leucocytes) and their patient-matched ratios (TLR) indicate large TLR-values in the areas of activated IDH-A expression (red areas) while low TLR were found in areas collecting genes upregulated in IDH-O and IDH-wt (dashed curve). This is in agreement with previous results showing that ALT-TMM (in IDH-A) usually associates with higher amounts of telomeric repeats compared with TEL-TMM (in IDH-wt and IDH-O) and thus with larger TLR-values [24]. Telomere length data (estimated from DNA seq) and patient matched expression data of LGG were taken from TCGA [24] and classified according to our subtypes and mapped to the LGG-SOM [8].

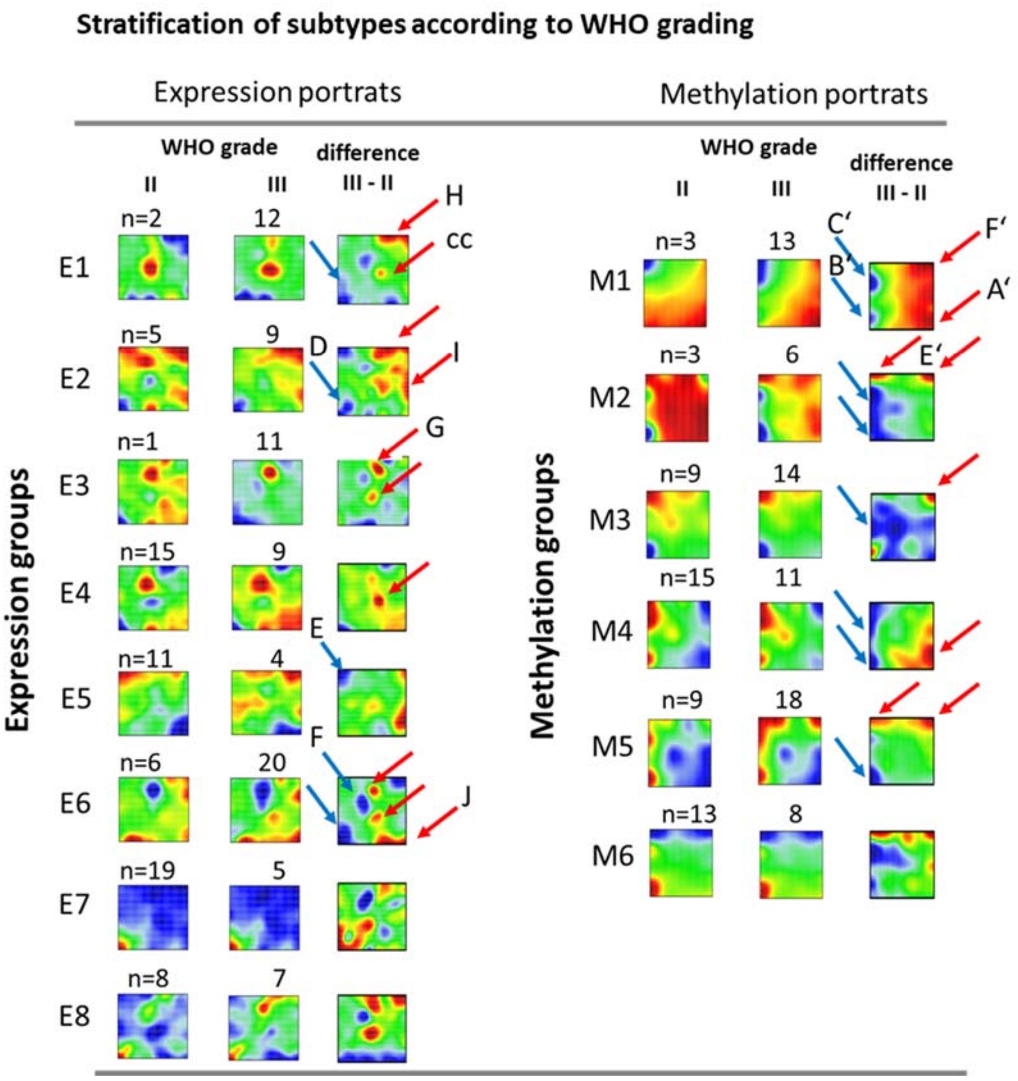

Figure S 19: Stratification of the E- and M-subtype portraits into mean portraits of WHO grades II and III tumors and of the difference between them. The arrows point to spots of increased (red) or decreased (blue) expression/methylation level in grade III compared to grade II gliomas in the difference portraits. Typically, cancer-related spots gain in expression in grade III gliomas, e.g. spot H in E1 and E2, spot I (translation) in E2 and spot G (inflammation) in E3. Spot E in E5, on the other hand however decays in grade III tumors indicating that activation of CT-genes is an early event in gliomagenesis. Spot cc (cell cycle) gains in almost all subtypes but especially in E6. Contrarily, the healthy brain signature (spot D) decreases in grade III together with the astrocyte-signature (spot F) compared with grade II (see, e.g., portraits of E6). Methylation of the *GPCR*- (spot B') and keratinocyte- (spot C') patterns decreases in grade III gliomas while GCIMP (spot D'), anti-GCIMP (spot F') and GCIMP-wt (spot A') gain methylation in grade III. Hence, as a general trend one finds that expression and methylation of cancer-related features increases in higher-grade tumors.

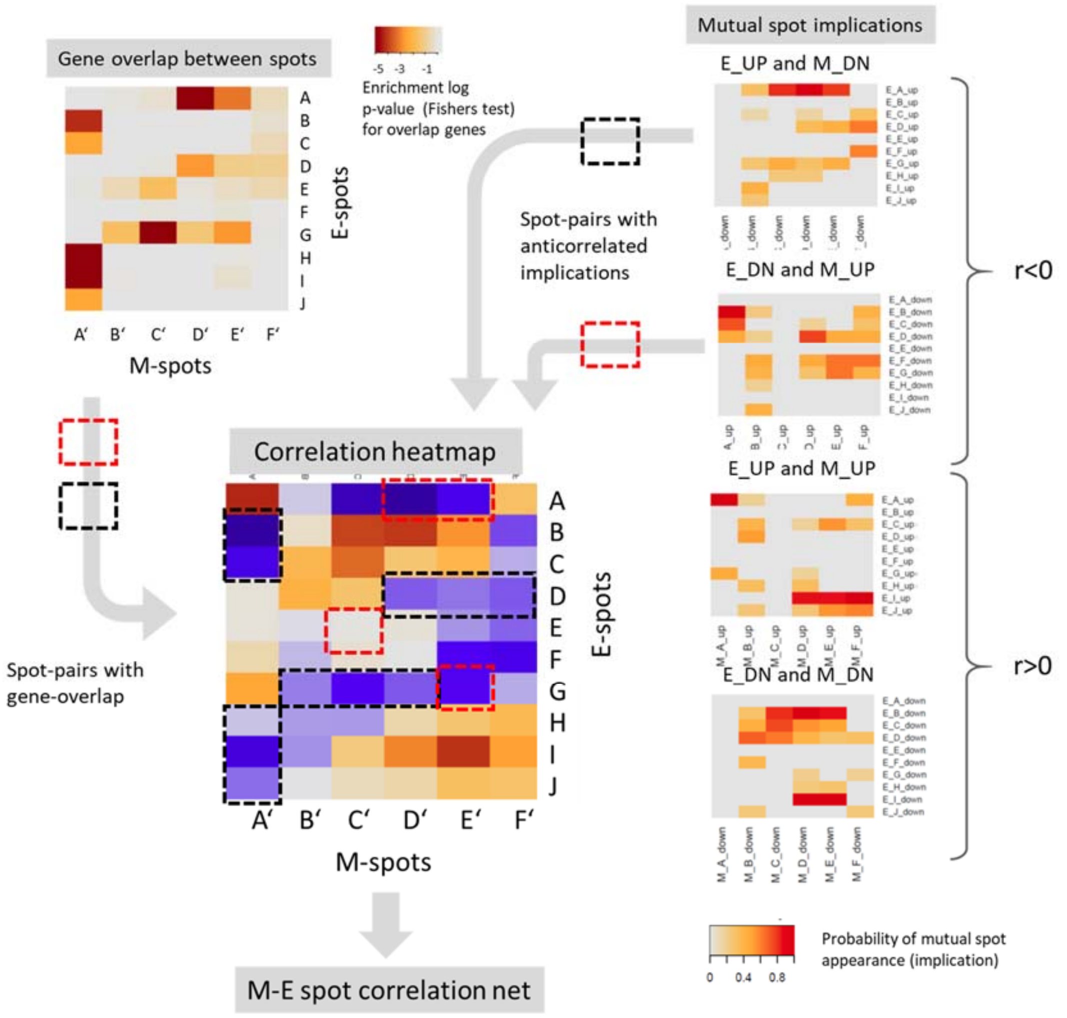

Figure S 20: Construction of the mutual network between expression and methylation spots: We first estimated three types of relations between the E- and M-spots and represented them as heatmaps: (i) the overlap of genes, (ii) the correlation between the expression and methylation profiles of the spots and (iii) the mutual spot implication (e.g. the probability to find a combination of spot-regulations in the SOM portraits of the patients) which consider the four types UP-DN, DN-UP, UP-UP and DN-DN between expression and methylation levels separately. The data show that spot combinations of non-negligible gene overlap show negatively correlated spot profiles with either dominating UP-DN or DN-UP combinations which finally were used to form the edges of the net.

Supplementary Tables

Table S 1: Functional enrichment of expression spots

Spot	short name	UP in	DN in	Gene sets [a] glioma [b]	epigenetics[c]	functional[d]	Chr[e]
A	CL-/PG-like	E1	E2-E6	Verhaak_CL-subtype, Reifenberger_GBM-wt, Gorovets_LGG-PG-subtype; Weller_LGG-gradeII-vs-III_DN	Noushmehr_GCIMP-genes, Fetal_TssA&TssP	BenPorath_cycling-genes; BP_cell-cycle; CC_extracellular_matrix , HM_EMT; HM-E2F2-targtes; HM_G2M-checkpoint	
B	PN-/EPL-like	E2, E4& E6	E1	Verhaak_PN-subtype; Gorovets_LGG-EPL-subtype;		BenPorath_SUZ12-targets; BP_nervous-system-development;	10
C	PN, oligo	E6& E7	E1& E3	Wirth_nervous-system, Weller_LGG-1p19qdel-vs-intact; Weller_LGG-A-vs-O_DN; Reifenberger_PN; Verhhaak_PN		Hebenstreit_low-TF, BenPorath_PRC2-targets; Benporath_SUZ12-targets; BenPorath_ES-with-H3K27me3; BP_nervous-system-development;	
D	NL-like/brain	E7	E1-E4	Reifenberger_PN-subtype	ReprPC_MidFrontal-lobe; TssP_mid_frantal-lobe; RA_neuronal-system	Wirth_Nervous-system, BenPoratth_ESC-wit-H3K27me3; BenPorath_SUZ12-targets; CC_synapse	
E	NB-like/testis	E5		Gorovets_LGG-NB-subtype	Hopp_Sturm-healthy-brain-methylation-UP	CC_hemoglobin-comples; MF_oxygen-binding; Wirth_testis, Jongeneel_testis; Martens_Tretinoin-response	19
F	astro	E4	E6	Weller_LGG-1p19q-codel-vs-intact_DN Weller_LGG-A-vs-O_UP; Lein_Astrocyte-markers	TssP_fetal-brain; TssA_fetal-brain; Hopp_Sturm_fetus-meth_UP;	LU_ageing-brain_UP, Bialock_Alzheimers__UP; LEE_BMP2-targets_UP; mature_astrocytes	1
G	MES-like; Immune response	E3	E6& E7	Reifenberger_GBM-CL&MES-subtypes; Donson_innate-immunity-associated-with-LTS;		BP_immune—response; BP_inflammatory-response; HM_interferon-gamma; HM_inflammatory-response	
H	PDGFRA asso-ciated	E2& E5	E4	Reifenberger_PNwt&MES_UP, Weller-LGG-A-vs-O	ReprPC_overlap-genes	BP_Transcription DNA-templated; CC_nucleoplasm; Shen_Smarxa2-targets_UP; Bialock_Alzheimers_disease_UP; LU_common-cancer-genes;	
I	Translation/ Oligo	E6	E1& E7	Verhaak_PN-subtype; , Weller_LGG-A-vs-O_DN	ReprPC_overlap-genes;	RE_translation;	10

J	EGFR-associated	E1-E4, E5	E5	Reifenberger_PNwt&CL_UP; EGFR	ReprPC_overlap-genes; Hopp-Sturm_fetal-brain_UP;	Senese_HDAC-targets; Wirth_immune-system; BP_transcription-DNAtemplated; CC_nucleus; Chaussabel_cytoskeleton	7

[a] gene sets with $p<10^{-7}$ (Fishers exact test) for enrichment in the resp. spot were considered; see Table S 3 for the full list of genes of the spots

[b] glioma expression signatures were taken from [5, 7, 25, 26, 27, 28, 29]

[c] glioma methylation signatures [30, 31, 32] and genes from chromatin states in healthy brain [33]

[d] literature sets from [34, 35, 36, 37, 38, 39, 40, 41, 42, 43, 44]; BP, CC, MF: Gene ontology terms biological process, cellular component and molecular function; HM: hallmarks of cancer [2]

[e] enriched genes from chromosomes

Table S 2: Functional enrichment of methylation spots

Spot	short name	UP in	DN in	Gene sets [a] glioma [b]	epigenetics[c]	functional[d]	Chr[e]
A‘	anti-GCIMP	M1&6	M3-5	Wirth_nervous-system; Verhaak_PN-subtype	Hopp-Sturm_fetus; Overlap_ReprPC; Overlap_HetRep	BP_transcription	19
B‘	GPCR	M4-6	M1-3		Hopp-Sturm_adult&fetus	MF_Olfactory_activity; KEGG_olfactory-transduction; CC_plasma-membrane; BP_immune-response	11
C‘	KER	M4-M6	M1&2		Hopp-Sturm_adult&fetus; Lembcke_CRC_CIMP-hypometh	CC_cornified_envelope; BP_keratinization; KEGG_olfactory-transduction; BP_G-protein-coupled-receptor-activity; CC_extracellular-space	
D‘	GCIMP	M2-5	M1&6	Reifenberger_IDH_wt-vs-mut_UP; Colman_survival-associated	Nousmethr_GCIMP; Hopp-Sturm_IDH; Christensen_hypermethylated-GradeII&III; Mif-Frontal_Lobe_ReprPC	Benporath_ES-with_H3K27me3; Benporath_SUZ12-targets; Benporath_EED-targets	
E‘	GCIMP-O	M5	M1&6		Hopp-Sturm_IDH; Christensen_hypermethylated-GradeII&III;	Benporath_ES-with_H3K27me3; Benporath_PRC2-targets; Lee_developmental-regulators	
F‘	GCIMP-wt (RTKII)	M1&5	M4&6		Hopp-Sturm_RTKII; Mid-Frontal-Lobe_TssP	Benporath_EED-targets; Benporath_PRC2-targets; BP_regfulation-of-transcription; Xie_senescence, Lee_developmental-regulators; BP_cell-differentiation; MF_sequence-specific-DNA_binding; CC_nucleus	

[a] gene sets with $p<10^{-76}$(Fishers exact test) for enrichment in the resp. spot were considered, see Table S 4 for the full list of genes of the spots

[b] glioma expression signatures; see [45], see also footnotes in Table S 1

[c] glioma methylation signatures [46, 47] and genes from chromatin states in healthy brain, see also footnotes in Table S 1

[d] BP, CC, MF: Gene ontology terms biological process, cellular component and molecular function; HM: hallmarks of cancer [2] and [34] and senescence methylation signature taken from [48]

[e] enriched genes from chromosomes

Table S 3: Gen lists of expression spot clusters A - J

Excel-Table

Table S 4: Genlists of methylation spot clusters A' – F'

Excel Table

References

1. Subramanian, A.; Tamayo, P.; Mootha, V. K.; Mukherjee, S.; Ebert, B. L.; Gillette, M. A., . . . Mesirov, J. P., Gene set enrichment analysis: A knowledge-based approach for interpreting genome-wide expression profiles. *Proceedings Of The National Academy Of Sciences Of The United States Of America* **2005,** 102, (43), 15545-15550.
2. Liberzon, A.; Birger, C.; Thorvaldsdóttir, H.; Ghandi, M.; Mesirov, Jill P.; Tamayo, P., The Molecular Signatures Database Hallmark Gene Set Collection. *Cell Systems* **2015,** 1, (6), 417-425.
3. Bindea, G.; Mlecnik, B.; Tosolini, M.; Kirilovsky, A.; Waldner, M.; Obenauf, A. C., Spatiotemporal dynamics of intratumoral immune cells reveal the immune landscape in human cancer. *Immunity.* **2013,** 39, 782 - 795.
4. Whitfield, M. L.; Sherlock, G.; Saldanha, A. J.; Murray, J. I.; Ball, C. A.; Alexander, K. E., . . . Botstein, D., Identification of Genes Periodically Expressed in the Human Cell Cycle and Their Expression in Tumors. *Molecular Biology of the Cell* **2002,** 13, (6), 1977-2000.
5. Weller, M.; Weber, R.; Willscher, E.; Riehmer, V.; Hentschel, B.; Kreuz, M., . . . Reifenberger, G., Molecular classification of diffuse cerebral WHO grade II/III gliomas using genome- and transcriptome-wide profiling improves stratification of prognostically distinct patient groups. *Acta Neuropathologica* **2015**, 1-15.
6. Brat, D. J.; Aldape, K.; Colman, H.; Figrarella-Branger, D.; Fuller, G. N.; Giannini, C., . . . Weller, M., cIMPACT-NOW update 5: recommended grading criteria and terminologies for IDH-mutant astrocytomas. *Acta Neuropathologica* **2020**.
7. Reifenberger, G.; Weber, R. G.; Riehmer, V.; Kaulich, K.; Willscher, E.; Wirth, H., . . . for the German Glioma, N., Molecular characterization of long-term survivors of glioblastoma using genome- and transcriptome-wide profiling. *International Journal of Cancer* **2014,** 135, (8), 1822-1831.
8. Binder, H.; Willscher, E.; Loeffler-Wirth, H.; Hopp, L.; Jones, D. T. W.; Pfister, S. M., . . . Loeffler, M., DNA methylation, transcriptome and genetic copy number signatures of diffuse cerebral WHO grade II/III gliomas resolve cancer heterogeneity and development. *Acta Neuropathologica Communications* **2019,** 7, (1), 59.
9. Venteicher, A. S.; Tirosh, I.; Hebert, C.; Yizhak, K.; Neftel, C.; Filbin, M. G., . . . Suvà, M. L., Decoupling genetics, lineages, and microenvironment in IDH-mutant gliomas by single-cell RNA-seq. *Science* **2017,** 355, (6332).
10. Reitman, Z. J.; Paolella, B. R.; Bergthold, G.; Pelton, K.; Becker, S.; Jones, R., . . . Beroukhim, R., Mitogenic and progenitor gene programmes in single pilocytic astrocytoma cells. *Nature Communications* **2019,** 10, (1), 3731.
11. Shaknovich, R.; Cerchietti, L.; Tsikitas, L.; Kormaksson, M.; De, S.; Figueroa, M. E., . . . Melnick, A., DNA methyltransferase 1 and DNA methylation patterning contribute to germinal center B-cell differentiation. *Blood* **2011,** 118, (13), 3559-3569.
12. Rajendran, G.; Shanmuganandam, K.; Bendre, A.; Mujumdar, D.; Goel, A.; Shiras, A., Epigenetic regulation of DNA methyltransferases: DNMT1 and DNMT3B in gliomas. *Journal of Neuro-Oncology* **2011,** 104, (2), 483-494.
13. J. Dabrowski, M.; Wojtas, B., Global DNA Methylation Patterns in Human Gliomas and Their Interplay with Other Epigenetic Modifications. *International Journal of Molecular Sciences* **2019,** 20, (14), 3478.
14. Cheng, T.; Xu, Y., Effects of Enhancer of Zeste Homolog 2 (EZH2) Expression on Brain Glioma Cell Proliferation and Tumorigenesis. *Medical science monitor : international medical journal of experimental and clinical research* **2018,** 24, 7249-7255.
15. de Vries, Nienke A.; Hulsman, D.; Akhtar, W.; de Jong, J.; Miles, Denise C.; Blom, M., . . . van Lohuizen, M., Prolonged Ezh2 Depletion in Glioblastoma Causes a Robust Switch in Cell Fate Resulting in Tumor Progression. *Cell Reports* **2015,** 10, (3), 383-397.

16. Mortimer, T.; Wainwright, E. N.; Patel, H.; Siow, B. M.; Jaunmuktane, Z.; Brandner, S.; Scaffidi, P., Redistribution of EZH2 promotes malignant phenotypes by rewiring developmental programmes. *EMBO reports* **2019,** 20, (10), e48155.
17. Maleszewska, M.; Kaminska, B., Deregulation of histone-modifying enzymes and chromatin structure modifiers contributes to glioma development. *Future Oncology* **2015,** 11, (18), 2587-2601.
18. Bian, E.-B.; Zong, G.; Xie, Y.-S.; Meng, X.-M.; Huang, C.; Li, J.; Zhao, B., TET family proteins: new players in gliomas. *Journal of Neuro-Oncology* **2014,** 116, (3), 429-435.
19. Delaunay, S.; Frye, M., RNA modifications regulating cell fate in cancer. *Nature Cell Biology* **2019,** 21, (5), 552-559.
20. Lian, H.; Wang, Q.-H.; Zhu, C.-B.; Ma, J.; Jin, W.-L., Deciphering the Epitranscriptome in Cancer. *Trends in Cancer* **2018,** 4, (3), 207-221.
21. Chai, R.-C.; Wu, F.; Wang, Q.-X.; Zhang, S.; Zhang, K.-N.; Liu, Y.-Q., . . . Kang, C.-S., m⁶A RNA methylation regulators contribute to malignant progression and have clinical prognostic impact in gliomas. *Aging* **2019,** 11, (4), 1204-1225.
22. Nersisyan, L.; Hopp, L.; Loeffler-Wirth, H.; Galle, J.; Loeffler, M.; Arakelyan, A.; Binder, H., Telomere Length Maintenance and Its Transcriptional Regulation in Lynch Syndrome and Sporadic Colorectal Carcinoma. *Frontiers in Oncology* **2019,** 9, (1172).
23. Barthel, F. P.; Wei, W.; Tang, M.; Martinez-Ledesma, E.; Hu, X.; Amin, S. B., . . . Verhaak, R. G. W., Systematic analysis of telomere length and somatic alterations in 31 cancer types. *Nature Genetics* **2017,** 49, 349.
24. Ceccarelli, M.; Barthel, Floris P.; Malta, Tathiane M.; Sabedot, Thais S.; Salama, Sofie R.; Murray, Bradley A., . . . Verhaak, R. G. W., Molecular Profiling Reveals Biologically Discrete Subsets and Pathways of Progression in Diffuse Glioma. *Cell* **2016,** 164, (3), 550-563.
25. Verhaak, R. G. W.; Hoadley, K. A.; Purdom, E.; Wang, V.; Qi, Y.; Wilkerson, M. D., . . . Hayes, D. N., Integrated Genomic Analysis Identifies Clinically Relevant Subtypes of Glioblastoma Characterized by Abnormalities in PDGFRA, IDH1, EGFR, and NF1. *Cancer Cell* **2010,** 17, (1), 98-110.
26. Gorovets, D.; Kannan, K.; Shen, R.; Kastenhuber, E. R.; Islamdoust, N.; Campos, C., . . . Huse, J. T., IDH Mutation and Neuroglial Developmental Features Define Clinically Distinct Subclasses of Lower Grade Diffuse Astrocytic Glioma. *Clinical Cancer Research* **2012,** 18, (9), 2490-2501.
27. Lein, E. S.; Hawrylycz, M. J.; Ao, N.; Ayres, M.; Bensinger, A.; Bernard, A., . . . Jones, A. R., Genome-wide atlas of gene expression in the adult mouse brain. *Nature* **2006,** 445, 168.
28. Donson, A. M.; Birks, D. K.; Schittone, S. A.; Kleinschmidt-DeMasters, B. K.; Sun, D. Y.; Hemenway, M. F., . . . Foreman, N. K., Increased Immune Gene Expression and Immune Cell Infiltration in High-Grade Astrocytoma Distinguish Long-Term from Short-Term Survivors. *The Journal of Immunology* **2012,** 189, (4), 1920-1927.
29. Wirth, H.; Löffler, M.; von Bergen, M.; Binder, H., Expression cartography of human tissues using self organizing maps. *BMC Bioinformatics* **2011,** 12, (1), 306.
30. Sturm, D.; Bender, S.; Jones, D. T. W.; Lichter, P.; Grill, J.; Becher, O., . . . Pfister, S. M., Paediatric and adult glioblastoma: multiform (epi)genomic culprits emerge. *Nat Rev Cancer* **2014,** 14, (2), 92-107.
31. Hopp, L.; Willscher, E.; Wirth-Loeffler, H.; Binder, H., Function Shapes Content: DNA-Methylation Marker Genes and their Impact for Molecular Mechanisms of Glioma. *Journal of Cancer Research Updates* **2015,** 4, (4), 127-148.
32. Noushmehr, H.; Weisenberger, D. J.; Diefes, K.; Phillips, H. S.; Pujara, K.; Berman, B. P., . . . Aldape, K., Identification of a CpG Island Methylator Phenotype that Defines a Distinct Subgroup of Glioma. *Cancer Cell* **2010,** 17, (5), 510-522.
33. Roadmap Epigenomics Consortium; Kundaje, A.; Meuleman, W.; Ernst, J.; Bilenky, M.; Yen, A., . . . Kellis, M., Integrative analysis of 111 reference human epigenomes. *Nature* **2015,** 518, (7539), 317-330.

34. Ben-Porath, I.; Thomson, M. W.; Carey, V. J.; Ge, R.; Bell, G. W.; Regev, A.; Weinberg, R. A., An embryonic stem cell-like gene expression signature in poorly differentiated aggressive human tumors. *Nat Genet* **2008,** 40, (5), 499-507.
35. Hebenstreit, D.; Fang, M.; Gu, M.; Charoensawan, V.; van Oudenaarden, A.; Teichmann, S. A., RNA sequencing reveals two major classes of gene expression levels in metazoan cells. *Mol Syst Biol* **2011,** 7.
36. Jongeneel, C. V.; Delorenzi, M.; Iseli, C.; Zhou, D.; Haudenschild, C. D.; Khrebtukova, I., . . . Vasicek, T. J., An atlas of human gene expression from massively parallel signature sequencing (MPSS). *Genome Research* **2005,** 15, (7), 1007-1014.
37. Martens, J. H. A.; Brinkman, A. B.; Simmer, F.; Francoijs, K.-J.; Nebbioso, A.; Ferrara, F., . . . Stunnenberg, H. G., PML-RARα/RXR Alters the Epigenetic Landscape in Acute Promyelocytic Leukemia. *Cancer Cell* 17, (2), 173-185.
38. Lu, T.; Pan, Y.; Kao, S.-Y.; Li, C.; Kohane, I.; Chan, J.; Yankner, B. A., Gene regulation and DNA damage in the ageing human brain. *Nature* **2004,** 429, (6994), 883-891.
39. Blalock, E. M.; Geddes, J. W.; Chen, K. C.; Porter, N. M.; Markesbery, W. R.; Landfield, P. W., Incipient Alzheimer's disease: Microarray correlation analyses reveal major transcriptional and tumor suppressor responses. *Proceedings of the National Academy of Sciences* **2004,** 101, (7), 2173-2178.
40. Lee, K. Y.; Jeong, J.-W.; Wang, J.; Ma, L.; Martin, J. F.; Tsai, S. Y., . . . DeMayo, F. J., Bmp2 Is Critical for the Murine Uterine Decidual Response. *Molecular and Cellular Biology* **2007,** 27, (15), 5468-5478.
41. Shen, H.; Powers, N.; Saini, N.; Comstock, C. E. S.; Sharma, A.; Weaver, K., . . . Knudsen, K. E., The SWI/SNF ATPase Brm Is a Gatekeeper of Proliferative Control in Prostate Cancer. *Cancer Research* **2008,** 68, (24), 10154-10162.
42. Lu, Y.; Yi, Y.; Liu, P.; Wen, W.; James, M.; Wang, D.; You, M., Common Human Cancer Genes Discovered by Integrated Gene-Expression Analysis. *PLOS one* **2007,** 2, (11), e1149.
43. Senese, S.; Zaragoza, K.; Minardi, S.; Muradore, I.; Ronzoni, S.; Passafaro, A., . . . Chiocca, S., Role for Histone Deacetylase 1 in Human Tumor Cell Proliferation. *Molecular and Cellular Biology* **2007,** 27, (13), 4784-4795.
44. Chaussabel, D.; Quinn, C.; Shen, J.; Patel, P.; Glaser, C.; Baldwin, N., . . . Pascual, V., A Modular Analysis Framework for Blood Genomics Studies: Application to Systemic Lupus Erythematosus. *Immunity* **2008,** 29, (1), 150-164.
45. Colman, H.; Zhang, L.; Sulman, E. P.; McDonald, J. M.; Shooshtari, N. L.; Rivera, A., . . . Aldape, K., A multigene predictor of outcome in glioblastoma. *Neuro-Oncology* **2010,** 12, (1), 49-57.
46. Binder, H.; Hopp, L.; Lembcke, K.; Wirth, H., Personalized Disease Phenotypes from Massive OMICs Data. In *Big Data Analytics in Bioinformatics and Healthcare*, Baoying, W.; Ruowang, L.; William, P., Eds. IGI Global: Hershey, PA, USA, 2015; pp 359-378.
47. Christensen, B. C.; Smith, A. A.; Zheng, S.; Koestler, D. C.; Houseman, E. A.; Marsit, C. J., . . . Wiencke, J. K., DNA Methylation, Isocitrate Dehydrogenase Mutation, and Survival in Glioma. *Journal of the National Cancer Institute* **2011,** 103, (2), 143-153.
48. Xie, W.; Kagiampakis, I.; Pan, L.; Zhang, Y. W.; Murphy, L.; Tao, Y., . . . Easwaran, H., DNA Methylation Patterns Separate Senescence from Transformation Potential and Indicate Cancer Risk. *Cancer Cell* **2018,** 33, (2), 309-321.e5.
